# Magnetic Field‐Driven Catalysis: Revealing Enhanced Oxygen Reactions in Li‐O_2_ Batteries Using Tailored Magnetic Nanocatalysts

**DOI:** 10.1002/advs.202505633

**Published:** 2025-06-25

**Authors:** Yimin Chen, Xin Hu, Min Hong, Yi Zhu, Yuyu Su, Ye Fan, Zhenxiang Cheng, John Bell, Baozhi Yu, Ying Ian Chen

**Affiliations:** ^1^ Institute for Frontier Materials Deakin University 75 Pigdons Road Waurn Ponds Victoria 3216 Australia; ^2^ Centre for Future Materials University of Southern Queensland Springfield 4300 Australia; ^3^ School of Engineering STEM College RMIT University Melbourne Victoria 3000 Australia; ^4^ Institute for Superconducting and Electronic Materials Australian Institute of Innovative Materials University of Wollongong Wollongong New South Wales 2500 Australia

**Keywords:** electrocatalysts, lithium oxygen batteries, magnetic catalysis, metal oxides, saturation magnetization

## Abstract

Lithium‐oxygen (Li‐O_2_) batteries offer immense promise for next‐generation energy storage technology due to their ultra‐high theoretical energy density. However, their adoption faces challenges like large overpotential and slow oxygen reaction kinetics. This study introduces a novel strategy that leverages custom‐designed magnetic nanocatalysts and external magnetic fields to boost electrochemical performance. Mn‐Co‐Fe oxide catalysts with adjustable magnetic properties is developed and demonstrated the correlation between the magnetism of the catalysts and the enhancement of battery performance. In the presence of an external magnetic field, the paramagnetic oxygen molecules experience a Kelvin force, while the Li^+^ ions are influenced by a Lorentz force. This accelerates their diffusion, significantly enhancing the kinetics of both the oxygen reduction and oxygen evolution reactions. The catalyst with the highest magnetization boosted specific capacity by 52.9% (from 8143 to 12 455 mAh g⁻¹) and significantly lowered the overpotential. This breakthrough underscores magnetic field‐driven catalysis as a crucial advancement in unlocking the full potential of Li‐O_2_ batteries, setting new benchmarks for energy storage technology.

## Graphical abstract

A series of Mn‐Co‐Fe oxides with different saturated magnetization (*M*_s_) are prepared to evaluate the enhancement catalytic properties of magnetic catalysts on Li‐O_2_ batteries (LOBs). A deep understanding of the intricate relationship between the magnetism of electrocatalysts and the corresponding performance of LOBs under the influence of magnetic fields is needed to fully unlock the power of magnetism in LOBs.

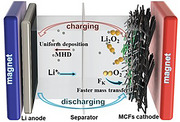

## 1 Introduction

Lithium oxygen batteries (LOBs) have been extensively investigated as one of the next‐generation energy storage systems owing to their high theoretical energy density of 3500 Wh kg^−1^.^[^ ^1^, ^2^ ^]^ However, the sluggish reaction kinetics of the oxygen reduction reaction (ORR) and the oxygen evolution reaction (OER), leading to the large overpotential and low energy conversion efficiency, are strongly limiting the large‐scale applications of LOBs.^[^ ^3^, ^4^, ^5^ ^]^ The conventional strategies focus on optimizing the internal components of Li‐O_2_ batteries, such as electrocatalysts, electrolytes, and soluble redox mediators. Besides the internal modifications, the application of an external physical field, such as the optical field, during the charging and discharging process of LOBs, has become a new trend in the development of high‐performance LOBs.^[^ ^6^, ^7^ ^]^ Introducing light to semiconductor electrocatalysts of LOBs can separate photoelectrons and holes, aiding in oxygen evolution and reduction.^[^ ^8^ ^]^ However, the shallow penetration of light and the confinement of electrode materials for semiconductors restrict the effectiveness of light‐assisted processes in these batteries. Magnetic fields, which are non‐destructive and free from penetration depth limitations, can effectively enhance oxygen reduction and evolution processes by speeding up reaction kinetics, modifying reaction pathways, and facilitating bubble release.^[^ ^9^, ^10^, ^11^ ^]^ Additionally, an exciting aspect is that magnetic transition metal oxides, commonly used as catalysts in Li‐O_2_ batteries, have inherent magnetic properties, presenting a promising opportunity to accelerate electrochemical processes in magnetic field‐assisted systems. Despite these encouraging prospects, the exact mechanisms by which magnetic electrocatalysts influence the performance of LOBs under a magnetic field remain under active investigation, marking a crucial frontier in this rapidly growing field of research.

Improving the kinetics of oxygen reduction and evolution reactions using magnetic catalysts in the presence of a magnetic field has demonstrated significant potential. It has been observed that applying a magnetic field significantly affects paramagnetic oxygen molecules, resulting in increased electron transfer rates during ORR.^[^ ^12^ ^]^ Similarly, the magnetic field‐enhanced OER process has also been proven in the water‐splitting reaction because the magnetic field favors the parallel alignment of oxygen radicals.^[^ ^13^, ^14^, ^15^, ^16^, ^17^ ^]^ These effects, whereby magnetic fields accelerate ORR and OER processes, can be attributed to a range of magnetic field‐induced phenomena, including the magnetothermal effect,^[^ ^18^ ^]^ spin polarization effect,^[^ ^17^ ^]^ magnetohydrodynamic (MHD) effect,^[^ ^19^, ^20^ ^]^ and electron energy state enhancement effect.^[^ ^12^ ^]^ Essentially, magnetic fields, mediated by magnetic materials, facilitate the alignment of electron spins, thereby expediting both OER and ORR processes. However, transitioning from magnetic field‐assisted OER and ORR to their implementation in LOBs entails addressing several key challenges. These encompass aspects such as product accumulation, the generation of reaction intermediates, and oxygen diffusion at the solid‐liquid interface. Furthermore, given that magnetism is an inherent property of materials, comprehending how the magnetic characteristics of electrocatalysts influence the performance of LOBs involving both discharge and charge processes under magnetic fields is imperative. To advance the development of superior catalysts for magnetic field‐assisted LOBs, it is crucial to investigate the intricate relationship between the magnetic properties of electrocatalysts and the corresponding performance of LOBs under the influence of magnetic fields, however, it has not been investigated so far.

Herein, we developed a series of Mn‐Co‐Fe oxides (MCFs) to serve as magnetic electrocatalysts for magnetic field‐assisted LOBs. The saturated magnetization (*M*_s_) of as‐prepared MCFs is successfully adjusted by controlling Mn and Co doping ratios in MCFs, where the *M*_s_ of MCF nanoparticles decreased with the increase of Mn and Co ratios in MCFs. Following this, LOBs with MCF catalysts were fabricated and their performance was assessed under the presence of a magnetic field. Furthermore, the influence of MCFs’ magnetism on LOBs performance was analyzed. Using Comsol Multiphysics simulation, the local magnetic field distribution of MCF catalysts with different *M*_s_ under an external field was calculated and used to explain the changes in the performance of LOBs. Density functional theory (DFT) calculations were conducted to investigate the improved performance of magnetic catalysts in a magnetic field. The results indicated that the magnetic field lowered the energy barrier for both the formation and decomposition of Li_2_O_2_. This optimization allows for enhanced overpotential and specific capacity in MCF‐catalyzed Li‐O_2_ batteries under the influence of a magnetic field. Our analysis revealed a positive correlation between *M*_s_ and the enhancement of LOB performance. Notably, the specific capacity of LOBs with MCF electrocatalysts increases by 52.9%, and the overpotential decreases by 108 mV by applying an external magnetic field. Consequently, the concept of magnetic field‐assisted LOBs offers an effective strategy for enhancing LOB systems.

## 2 Results and Discussion

**Figure 1:**
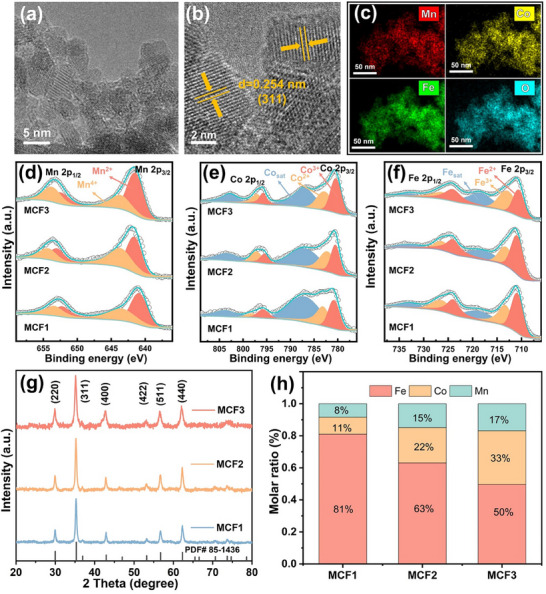
Material characterization of MCF catalysts. a) TEM, b) HR‐TEM, c) EDS mapping of MCF1, XPS spectra of d) Mn 2p. e) Co 2p, and f) Fe 2p, g) XRD patterns of investigated MCF catalysts, h) ICP‐OES analysis of MCF1, MCF2, and MCF3.

A series of magnetic electrocatalysts was produced by doping Mn and Co into the spinel‐structured Fe_3_O_4_ with a hydrothermal method using different molar ratios of MnCl_2_, CoCl_2_, and FeCl_3_ precursors.^[^ ^21^ ^]^ The obtained products with the precursor ratios of 1:1:8, 2:2:6, and 3:3:4 are named MCF1, MCF2, and MCF3, respectively. The morphologies and chemical compositions of the MCFs were analyzed with transmission electron microscopy (TEM). The TEM image of the prepared MCF1 shows a size of 6 ± 1 nm in **Figure** **1**a, and the HR‐TEM image of MCF1 (Figure 1b) displays the explicit lattice fringe spacing of 0.254 nm, corresponding to the (311) planes of Fe_3_O_4_.^[^ ^22^ ^]^ The EDS mapping and HAADF‐STEM show the uniform distribution of Mn, Co, Fe, and O atoms in MCF1 nanoparticles (Figure 1c; Figure S1, Supporting Information). The other two electrocatalysts, MCF2 and MCF3, show similar morphologies which are also confirmed with TEM images (Figure S2, Supporting Information). X‐ray photoelectron spectroscopy (XPS) measurements were used to examine the chemical composition and bonding states of the obtained MCF catalysts (Figure S3, Supporting Information). In Figure 1d, the Mn 2p spectra of MCF catalysts are depicted. Specifically, the Mn 2p_1/2_ peak at 640.35 eV and the Mn 2p_3/2_ peak at 651.59 eV can be ascribed to Mn^2+^.^[^ ^23^ ^]^ Furthermore, the Mn 2p_1/2_ peak at 643.19 eV and the Mn 2p_3/2_ peak at 652.89 eV correspond to Mn^4+^.^24^ Mn^2+^ and Mn^4+^ can also be found in the spectra of MCF2 and MCF3. Additionally, Figure 1e reveals that the Co 2p spectra of MCFs exhibit spin‐orbit peak splitting, where the Co 2p_3/2_ peak is located at 780.54 eV, and the Co 2p_1/2_ peak is found at 796.46 eV.^[^ ^25^, ^26^ ^]^ These peaks can be further divided into their respective components, representing Co^3+^ and Co^2+^. Moreover, the intensity of Mn 2p and Co 2p increases as the Mn and Co doping levels increase. The high‐resolution Fe 2p spectra of the obtained MCF (Figure 1f) exhibit the typical spin‐orbit splitting of Fe 2p_3/2_ and Fe 2p_1/2_, accompanied by corresponding satellite peaks.^[^ ^27^ ^]^ Furthermore, XRD patterns (Figure 1g) confirm the reverse spinel structures of the three MCFs, with all diffraction peaks matching the characteristic peaks of Fe_3_O_4_ (PDF# 85‐1436). To assess the relative metal concentration and doping levels of the MCFs, inductively coupled plasma‐optical emission spectroscopy (ICP‐OES) was employed. The results (Figure 1h) reveal that the Mn, Co, and Fe molar ratios in the catalysts, corresponding to MCF1, MCF2, and MCF3, are 8:11:81, 15:22:63, and 17:33:50, respectively. The results indicate that a well‐controlled ratio of Mn and Co in MCF catalysts was successfully obtained.

**Figure 2:**
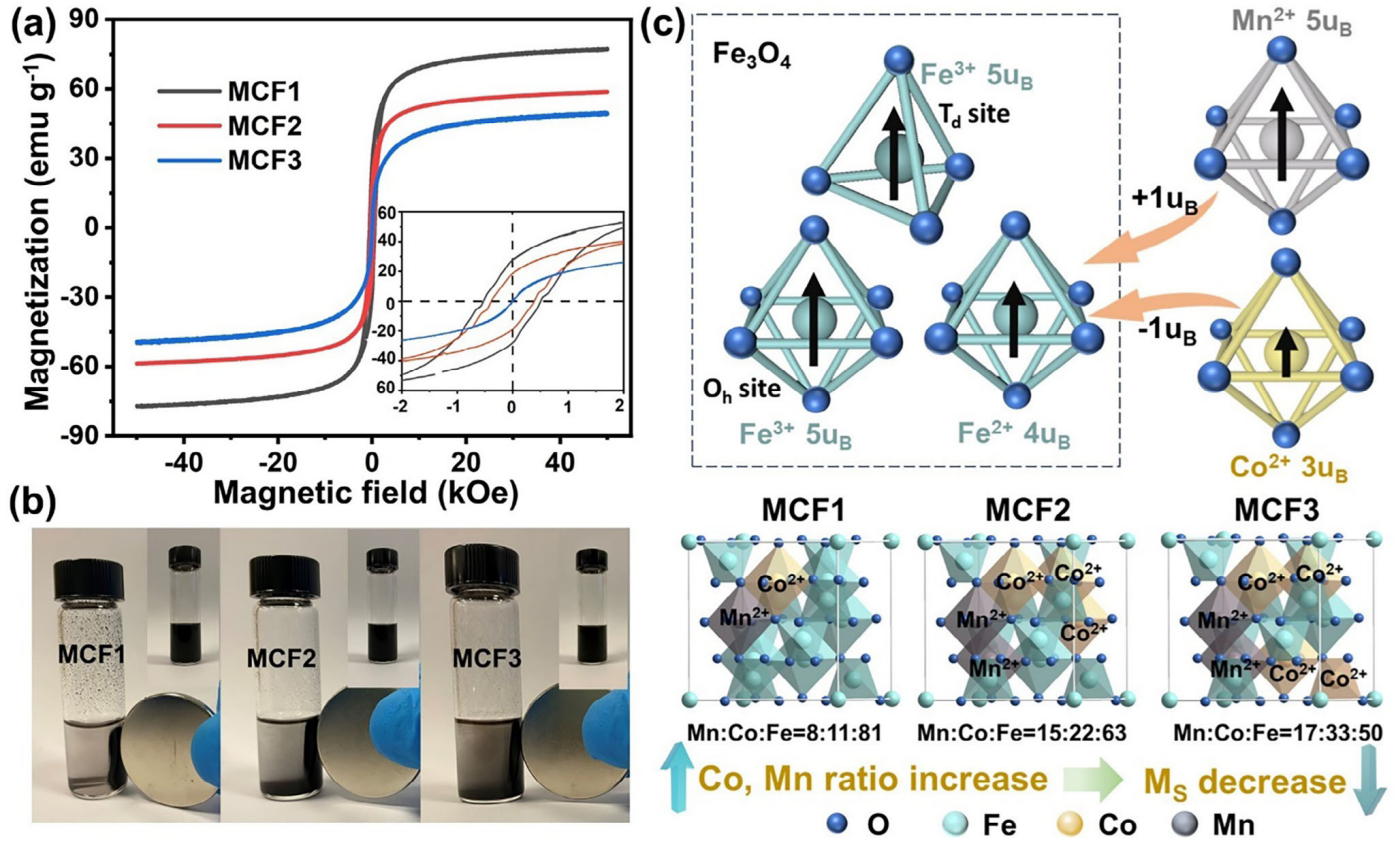
a) Magnetic hysteresis loops of MCFs measured at 300 K (inset is the magnified MH loop around H=0). b) Optical photograph of MCF1, MCF2, and MCF3 suspension with and without magnet field. c) Schematic illustration of the relationship between MCF magnetization and the doping ratio.

The magnetic properties of MCFs were analyzed by Vibrating Sample Magnetometer (VSM) with a field up to 5 T (Tesla). **Figure** **2**a shows the field‐dependent magnetization (M‐H) curves of MCF nanoparticles at 300 K. The magnetizations of MCF1, MCF2, and MCF3 at 5 T are 75, 58, and 49 emu g^−1^, respectively. The magnetization of the MCFs was found to be decreased as Mn and Co doping ratios increased in the MCFs. In the enlarged image in Figure 2a, the MCF3 has the lowest coercivity and remanent moment. The value of coercivity and remanence order is MCF1 > MCF2 > MCF3. To further corroborate the findings, we conducted a demonstration using a rare‐earth permanent magnet (Figure 2b). Initially, we dispersed MCF powders in ethanol to create a uniform suspension. When the magnet was brought close to the suspension, the powder accumulated in its vicinity. Notably, MCF1 powder exhibited a more significant accumulation near the magnet, resulting in a discernible suspension, while MCF2 and MCF3 showed relatively less powder collecting near the magnet. This observation supports the order of magnetization intensity as MCF1 > MCF2 > MCF3. Figure 2c provides a visual representation of how the ratios of Mn and Co in the MCFs influence their magnetization. To understand this, it is essential to consider the spinel structure of these MCF nanoparticles. This structure consists of an oxygen atom lattice arranged in a face‐centered cubic packing, with tetrahedral (Td) and octahedral sites (Oh).^[^ ^28^, ^29^ ^]^ The Co distributes across both the Td and Oh sites. While the Mn ions are predominating at the Oh sites. In contrast to [Fe^3+^]_d_[Fe^2+^Fe^3+^]_Oh_O_4_, the Mn^2+^ dopant can replace Fe^2+^ to provide a magnetic moment of 1 μB (Bohr magneton) per unit of the formula. Fe^2+^ can also be replaced with Co^2+^, which reduces the magnetic moment by 1 μB per unit of the formula. (Figure S4, Supporting Information) As we increase the ratio of Mn and Co doping, Co tends to occupy more sites compared to Mn in the MCF catalysts. Consequently, the overall magnetic moment gradually diminishes, going from 75 emu g^−1^ for MCF1 to 49 emu g^−1^ for MCF3. This trend clarifies the magnetic properties of the prepared MCFs, establishing the order as MCF1 > MCF2 > MCF3 in terms of their *M*_s_.

**Figure 3:**
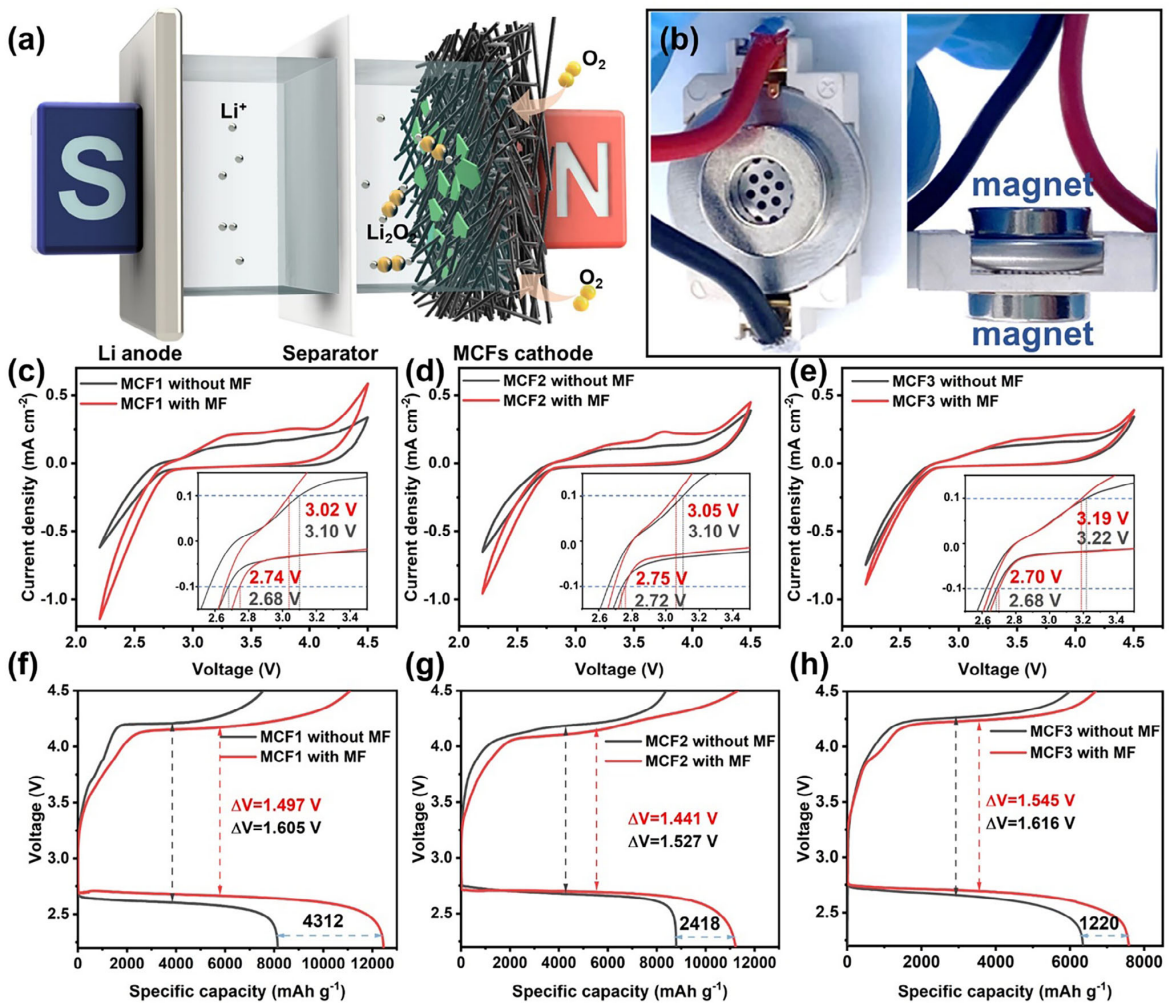
a) Schematic and b) photograph of LOBs with applied external magnetic fields. Cyclic voltammetry (CV) curves of c) MCF1, d) MCF2, and e) MCF3 cathodes with and without magnetic field at a scan rate 0.5 mV s^−1^ with the voltage window of 2.2–4.5 V. The full discharge/charge curves of f) MCF1, g) MCF2, and h) MCF3 with and without the magnet field.

To investigate the impact of MCF catalysts' magnetic properties on LOB performance under a magnetic field, we constructed coin cells with MCF catalysts and magnets on both sides (**Figure** **3**a,b; Figure S5, Supporting Information). The magnetic field strength applied was ≈120 mT. Initially, we conducted CV tests to evaluate the electrocatalytic activity of MCF‐catalyzed LOBs with and without a magnetic field. These tests were carried out at a scanning speed of 0.5 mV s^−1^ within a potential range of 2.2–4.5 V (vs Li/Li^+^), and the results are shown in Figure 3c–e. The onset potentials (E_onset_) of ORR and OER in the cells are defined as the voltage at which the current density reaches ±0.1 mA cm^−2^.^[^ ^30^, ^31^ ^]^ In Figure 3c, MCF1 displayed E_onset_ values of 2.68 V for ORR and 3.10 V for OER without a magnetic field. However, with the application of a magnetic field, MCF1 exhibited an increased ORR E_onset_ of 2.74 V and a decreased OER E_onset_ of 3.02 V, suggesting a reduction in reaction barriers.^[^ ^32^ ^]^ Similarly, Figure 3d,e demonstrates that MCF2 and MCF3 also experienced increased ORR Eonset and decreased OER Eonset when a magnetic field was applied.

To further evaluate the performance of the MCF‐catalyzed LOB with/without the external magnetic field, we conducted galvanostatic discharge/charge tests on the obtained cells with/without an external magnetic field at a current density of 200 mA g^−1^ (Figure 3f–h). Specific capacity calculations were based on the mass of MCF electrocatalysts in the cell. Notably, the introduction of a magnetic field led to significant enhancements. Specifically, the specific capacity of MCF1‐catalyzed LOBs increased from 8143 to 12455 mAh g^−1^, with the overpotential decreasing from 1.605 to 1.497 V (Figure 3f). Similarly, MCF2‐based LOBs demonstrated increased specific capacity, rising from 8797 to 11215 mAh g^−1^, and a decreased overpotential from 1.527 to 1.441 V (Figure 3g). Figure 3h revealed that MCF3‐catalyzed LOBs also benefited from the magnetic field, exhibiting an increased specific capacity of 1220 mAh g^−1^ and a decreased overpotential from 1.616 to 1.545 V. To elucidate the intricate interplay between material magnetism and the augmentation of LOBs performance under the influence of a magnetic field, a comprehensive summary of changes in ORR/OER onset potential, specific capacity, and overpotential in MCF catalyst‐based Li‐O_2_ cells, both with and without a magnetic field (Figure S6–S8, Supporting Information). In Figure S6 (Supporting Information), a clear trend emerges: the ORR onset potentials of cells featuring MCF1, MCF2, and MCF3 increased by 0.06, 0.03, and 0.02 V, respectively, when a magnetic field was applied. Concurrently, the OER onset potentials exhibited reductions of 0.08, 0.05, and 0.03 V, respectively. Notably, these shifts corresponded to the order of *M*_s_ in the MCFs, with MCF1, boasting the highest *M*_s_, experiencing the most substantial improvement among the three catalysts. Specific capacity and overpotential changes of the cells are depicted in Figures S7 and S8 (Supporting Information). Specifically, the cell with MCF1 witnessed a remarkable 52.9% increase in specific capacity compared to its non‐magnetic field counterpart. In contrast, MCF2 and MCF3 exhibited increases of 27.5% and 19.2%, respectively. As for overpotential reduction, MCF1 led with a 0.108 V decrease, followed by MCF2 at 0.086 V, and MCF3 at 0.071 V. Meanwhile, the effects of different magnetic field intensities on MCF1 catalyzed‐LOB performance were evaluated. As shown in Figure S9 (Supporting Information), when the magnetic field intensities are 0, 50, 120, and 300 mT, the specific capacity of LOBs at 200 mAg^−1^ is 8143, 9814, 12455, and 13416 mAh g^−1^, respectively. When the magnetic field strength increases from 120 to 300 mT, the specific capacity increases by 961 mAh g^−1^, which is less than the change when the magnetic field strength increases from 50 to 120 mT. This is because the MCF1 is close to complete magnetization at 300 mT. The magnetic field no longer influences the material's behavior in a way that significantly enhances its catalytic activity. Furthermore, the cycling performance for MCF cathodes with/without the magnetic field at a current density of 500 mA g^−1^ with a limited specific capacity of 500 mAh g^−1^ is illustrated in Figure S10 (Supporting Information). Clearly, with the help of the magnetic field, the MCF1‐based LOBs have cycling stability of more than 200 h, which surpasses that of those without the magnetic field. Meanwhile, the cycle stability of MCF2 and MCF3 was also improved with the magnetic field (Figure S10b,c, Supporting Information). These demonstrated that the magnetic field extends the cycling lifespan of MCF‐based LOBs. Based on these results, we can draw conclusions that the performances of magnetic electrocatalyst‐based LOBs can be enhanced by an external magnetic field and the enhancement is directly proportional to the electrocatalysts’ *M*_s_.

**Figure 4:**
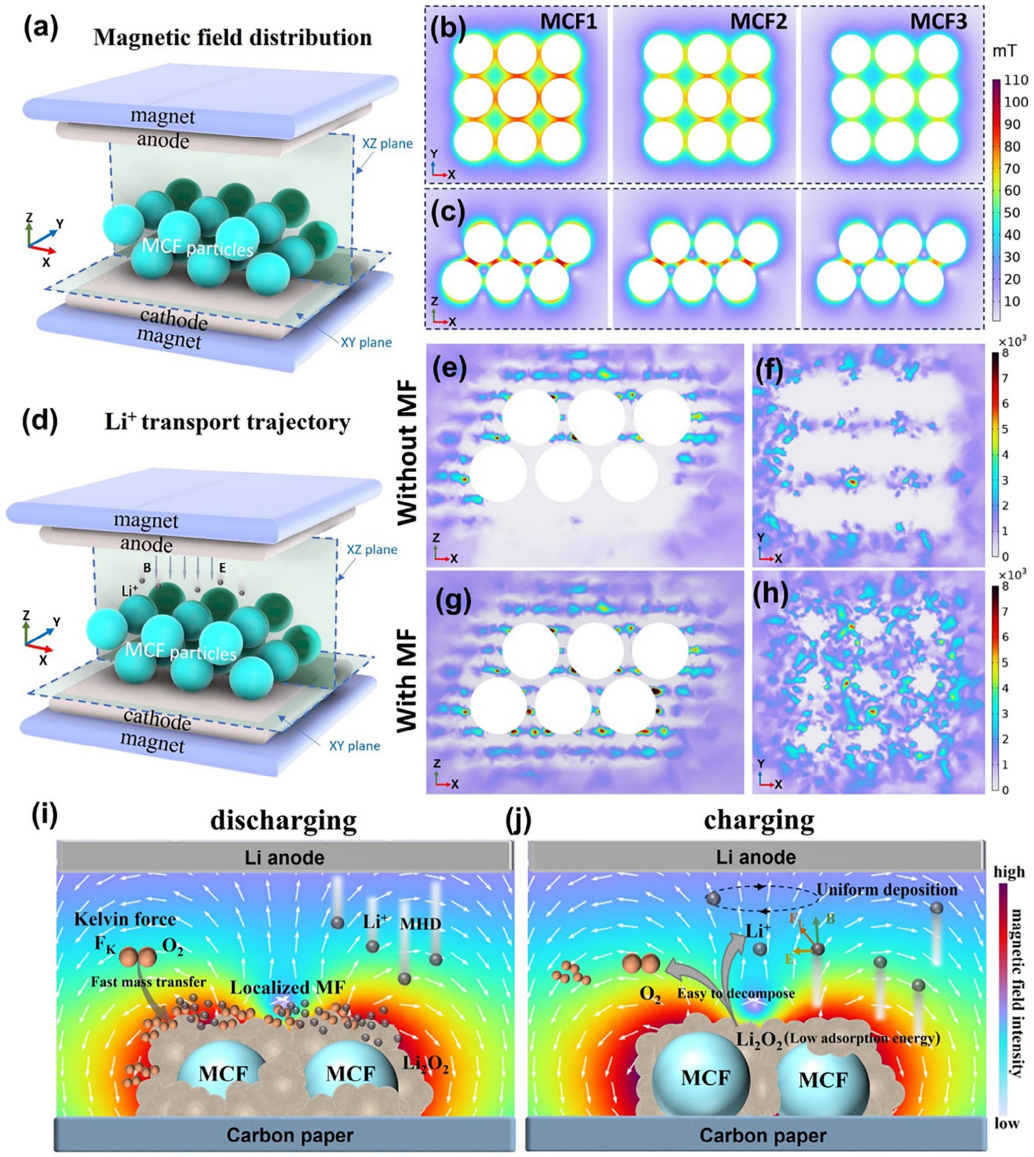
a) The schematic for the magnetic field distribution simulation of MCF electrodes after magnetization. b) XY plane and c) XZ plane. d) The schematic of Li^+^ transport trajectory without a magnetic field (MF) and with MF. The Li^+^ distribution on e) XZ plane and f) XY plane without MF, g) XZ plane, and h)XY plane with MF. Schematic illustration of magnetic catalysts assisted LOBs with magnetic field i) discharging process and j) charging process.

In an attempt to deeply explore the mechanism of magnetic properties‐dependent enhancement of the performance of LOBs, the magnetic fields around MCF particles in the LOBs need to be clearly understood. Therefore, the magnetic field distribution in the MCF‐catalyzed LOBs is simulated with COMSOL Multiphysics software. As shown in **Figure** **4**a, a pair of permanent magnets is applied on the cathode and the anode to provide a static magnetic field (Figure S11, Supporting Information). The results of the magnetic field distribution are extracted in the XY plane (Figure 4b) and XZ plane (Figure 4c). As shown in Figure 4b, the induced magnetic fields are generated around the MCF particles, while the intensity of the induced field of MCF1 is higher than that of MCF2 and MCF3. This is particularly evident along the *z*‐axis, where MCF1 demonstrates a stronger induced magnetic field. Based on these results, it can be found that the intensity of these fields is closely aligned with the *M*_s_ of the MCFs, implying that higher *M*_s_ values result in stronger magnetic induction by the particles. Additionally, the gradient of the induced magnetic fields is another important factor that may affect electrochemical reactions, so the magnetic field gradient (∇B) near nanoparticles was calculated and analyzed (Figure S12, Supporting Information). The value of ∇B around MCF1 nanoparticles reaches 7.1 × 10^4^ T m^−1^, the highest among these three MCFs. Meanwhile, the value of ∇B around MCF2 (6.5 × 10^4^ T m^−1^) is also higher than MCF3 (5.6 × 10^4^ T m^−1^), indicating a correlation between increased ∇B values and higher *M*_s_ values of the MCF catalysts.

After gaining insights into the magnetic fields in LOBs, we further investigated the transportation of Li⁺ ions near MCF particles under these fields. Using simulations, we compared Li⁺ ion trajectories with and without the influence of a magnetic field. As depicted in Figure 4d and Figure S11 (Supporting Information), the model includes static magnetic poles positioned on either side of the MCF particles, with Li⁺ ions released from the anode. The cross‐sectional diagrams in Figure 4e–h reveal distinct Li⁺ ion distributions after 10 ns of simulation time. In the absence of a magnetic field, Figure 4e,f shows that only a few Li⁺ ions manage to reach below the particles, indicating limited ion access to this region due to obstruction by the MCF particles. However, when both electric and magnetic fields are applied (Figure 4g), Li⁺ ions are more evenly distributed across the XZ plane, with a notably higher concentration around the second layer of MCF particles. Figure 4h further illustrates that under combined fields, Li⁺ ions bypass the lower MCF particles, suggesting that the Lorentz force (F_L_) in the gradient magnetic field facilitates their movement toward the MCF particles' vicinity. A comparative analysis of MCF2 and MCF3 (Figure S13, Supporting Information) reveals similar Li⁺ ion distribution patterns, while MCF1 exhibits a significantly higher concentration of Li⁺ ions. This observation aligns with the higher magnetic field intensity around MCF1, confirming that catalysts with greater *M*_s_ generate stronger magnetic fields, resulting in an increased Li⁺ concentration near the particles. In addition to influencing Li⁺ transport, the magnetic field also affects other reactants involved in the discharging/charging process, such as paramagnetic O_2_ molecules. These species experience the **Kelvin force** (F_K_), which accelerates their diffusion within the gradient magnetic field. This force is mathematically expressed as $F_{K}=\frac{1}{2u_{0}}C\chi_{m}\nabla B^{2}$, where μ_0_ stands for the permeability of free space, ∇B for the magnetic field gradient, χ_m_ for the molar magnetic susceptibility, and c for the concentration of the electroactive species in bulk.^[^ ^33^ ^]^ The F_K_ accelerates the mass transfer of O_2_ molecules, which have a permanent magnetic moment, thus improving oxygen reduction and evolution kinetics. Additionally, when the magnetic field gradient (∇B) is orthogonal to the concentration gradient (∇c), the reduction in diffusion layer thickness enhances the delivery of reactants to the electrode, increasing the limiting current.^[^ ^33^, ^34^ ^]^ The enhanced capacity of MCF‐catalyzed LOBs can be attributed to this F_K_ effect, which is stronger near particles with higher *M*_s_ values, allowing more reactants to reach the catalyst surface. This increase in reactant availability directly correlates with the observed rise in discharge capacity, particularly for MCF1. For the discharge process (Figure 4i), the applied magnetic field magnetizes the magnetic catalyst, creating a localized magnetic field that accelerates the transport of oxygen and lithium ions. This promotes the growth of Li_2_O_2_ in a flower‐like structure (Figures S14 and S15, Supporting Information), thereby increasing the battery capacity. Additionally, electrons are influenced by the magnetic field, which induces the degeneracy of unpaired electron spins, elevating electron energy states and reducing the net enthalpy of the activation barrier for electron‐transfer reactions, termed magnetic enhanced electron transfer (MEET).^[^ ^35^, ^36^ ^]^ As for the charging process (Figure 4j), flower‐like Li_2_O_2_ can be evenly distributed on the electrode surface, preventing pore blockage and improving the utilization of active sites. It is more easily oxidized and decomposed during the charging process, improving reversibility. In addition, Li^+^ ions migrate from the cathode to the lithium anode through the electrolyte. Upon approaching the MCFs, the magnetic field alters their path, provoking electrolyte convection and triggering the MHD effect, which induces spiral fluid dynamics.^[^ ^37^, ^38^, ^39^, ^40^, ^41^ ^]^ Li^+^ ions are affected by the MHD effect, which is more uniform when deposited on the anode (Figure S16, Supporting Information), reducing the formation of dendrites, and concurrently lowering the nucleation potential for lithium ions.^[^ ^42^, ^43^ ^]^ This corresponds well to the cell overpotential reduction order: MCF1>MCF2>MCF3. Overall, the higher the magnetization of MCF oxides, the higher the improvement of their catalyzed LOBs' performance.

**Figure 5:**
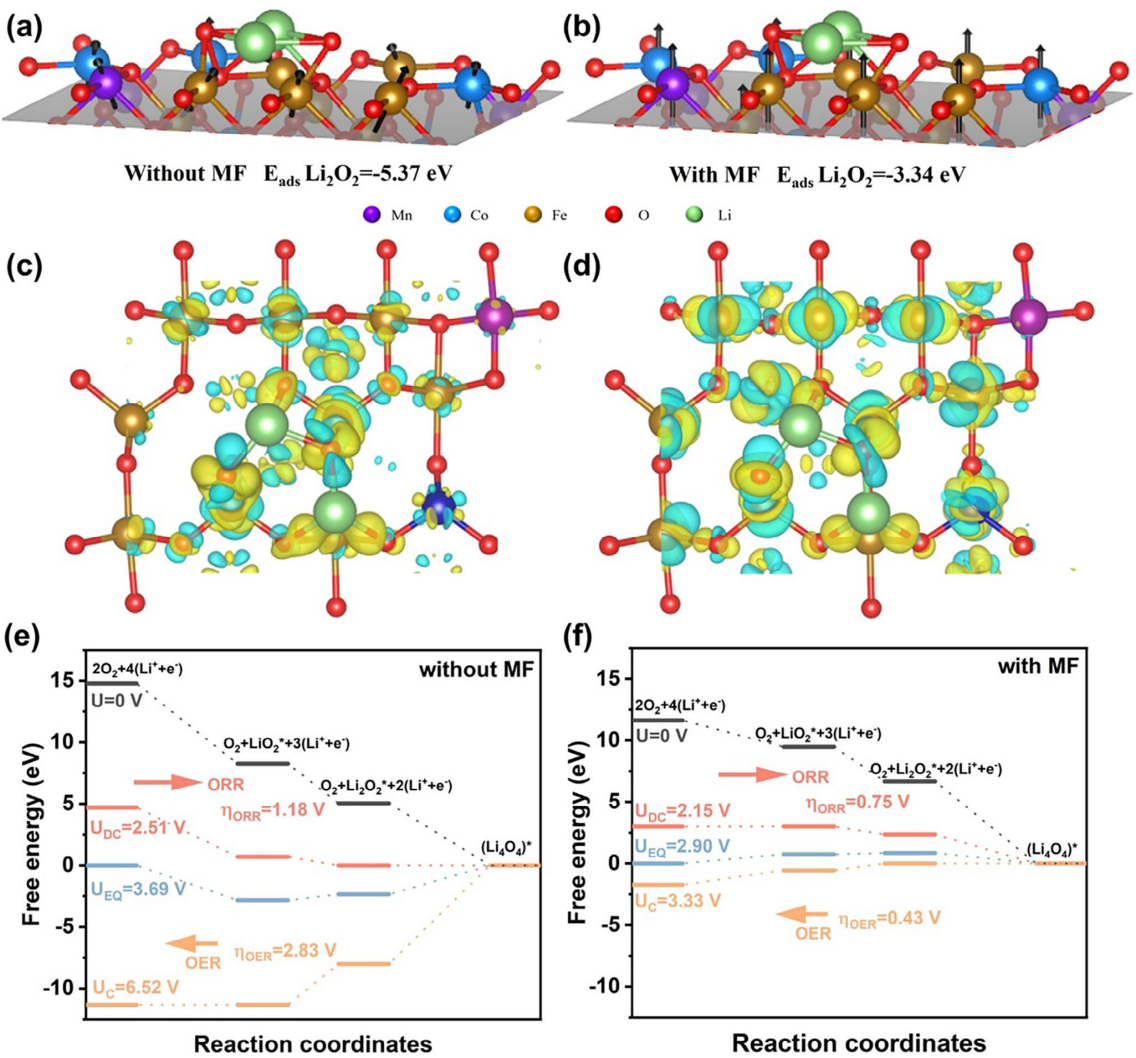
Calculated adsorption energies of Li_2_O_2_ on the MCF surface a) without and b) with magnetic field; Charge density difference plots of Li_2_O_2_* on (110) plane c) without and d) with magnetic field; The Gibbs free energy profiles at zero, equilibrium, and charge potentials for MCF cathodes without magnetic field e) and with magnetic field f).

In addition to explaining the enhancement of battery capacity by the magnetic field from the perspective of mass transfer, it can also be explained from the energy perspective, considering the impact of the magnetic field on the LOBs' overpotential. We investigated the adsorption energies of key intermediates and the free energy diagrams for MCF catalysts with and without a magnetic field. In general, the fundamental three‐step reaction processes are outlined for the oxygen electrode reactions in LOBs: (1) 4(Li^+^ + e^‐^) + O_2_ ↔ LiO_2_^∗^ + 3(Li^+^ + e^‐^), (2) LiO_2_^∗^ +3(Li^+^ + e^‐^) ↔ Li_2_O_2_^∗^ + 2(Li^+^ +e^‐^), and (3) Li_2_O_2_^∗^ + 2(Li^+^ + e^‐^) + O_2_ ↔ (Li_4_O_4_^∗^), in which the asterisk represents the absorbed species on the surface of the electrodes (Figure S17, Supporting Information). The energetically optimized adsorption interfaces of Li_2_O_2_ without and with a magnetic field are shown in **Figure** **5**a,b. As shown in Figure 5a, the MCF facet shows the strong binding interactions of −5.37 eV with the Li_2_O_2_ monomer without a magnetic field, which indicates that although the pristine MCF component easily absorbs Li_2_O_2_, the decomposition process needs to overcome larger energy barriers to realize the recharging.^[^ ^44^ ^]^ In contrast, when a magnetic field is applied, the adsorption energy decreases to −3.34 eV (Figure 5b). It means that the decomposition of Li_2_O_2_ needs small energy barriers, resulting in lower charge overpotential. From the charge density difference plots shown in Figure 5c,d, and Figure S18 (Supporting Information) (yellow and blue contours represent the increased and decreased electron density, respectively), it is obvious that the electron transfer of Li_2_O_2_ with the magnetic field is much higher than that without the magnetic field. More charge transfer can facilitate the efficient decomposition of discharge products during the charging process, thereby reducing the OER potential.^[^ ^45^ ^]^ The Gibbs free energy diagrams of oxygen electrode reactions on MCF are displayed in Figure 5e,f. The discharge and charge overpotentials are defined as 𝜂_ORR_ = U_0_‐U_DC_ and 𝜂_OER_ = U_C_‐U_0_, respectively, where U_0_, U_DC_, and U_C_ are equilibrium, discharged, and charged potentials, respectively. As shown in Figure 5e, the MCF catalyst shows maximal overpotentials of 𝜂_ORR_ = 1.18 V and 𝜂_OER_ = 2.83 V during the ORR and OER process, respectively. When the magnetic field is added to the MCF‐catalyzed LOBs, the ORR and OER overpotentials on MCF are reduced to 0.75 and 0.43 V, respectively, revealing significantly promoted electrocatalytic activity of the magnetic catalysts toward oxygen redox reactions in LOBs.

## 3 Conclusion

We introduce a novel approach for improving LOB's performance by combining external magnetic fields with MCF nanocatalysts of tunable magnetic properties. The research demonstrates a direct correlation between the *M*_s_ of the catalysts and enhancements in ORR and OER. Under the influence of magnetic fields, paramagnetic oxygen, and Li⁺ ions experience Kelvin and Lorentz forces, respectively, leading to faster mass transfer, improved reaction kinetics, and reduced overpotential. This results in up to a 52.9% increase in specific capacity and a notable 108 mV reduction in overpotential. Supported by simulations and calculations, the study reveals that magnetic fields lower energy barriers for Li_2_O_2_ formation and decomposition, addressing key performance limitations of Li‐O_2_ batteries. This work pioneers magnetic field‐driven catalysis in batteries, offering a scalable, innovative strategy to boost energy storage efficiency, capacity, and stability, marking a significant advancement toward next‐generation battery technologies.

## Conflict of Interest

The authors declare no conflict of interest.

## Author Contributions

B.Z. and Y.‐I.C. supervised the project. B.Z. and Y.C. conceived the idea and designed the experiments. Y.C. performed all the experimental work. Z.C. carried out the VSM experiments and manuscript editing. Y.S. performed the XPS measurements. Y.Z. and X.H. assisted in catalyst preparation and plotted the Figs. Y.F. contributed to data analysis and manuscript editing. M.H. and J.B. contributed to DFT calculations. B.Z., Y.C., and Y.I.C wrote the manuscript.

## Supporting information



Supporting Information

## Data Availability

The data that support the findings of this study are available from the corresponding author upon reasonable request.
