# Supplementary material for: Magnetic Field‐Driven Catalysis: Revealing Enhanced Oxygen Reactions in Li‐O2 Batteries Using Tailored Magnetic Nanocatalysts
Source: Adv Sci (Weinh). 2025 Jun 25;12(34):e05633. doi: 10.1002/advs.202505633 (PMC12442637; doi:10.1002/advs.202505633)
Supplement: Supplementary file 1 — Supporting Information [file ADVS-12-e05633-s001.docx]

Supporting Information

**Magnetic Field-Driven Catalysis: Revealing Enhanced Oxygen Reactions in Li-O_2_ Batteries Using Tailored Magnetic Nanocatalysts**

*Yimin Chen, Xin Hu, Min Hong, Yi Zhu, Yuyu Su, Ye Fan, Zhenxiang Cheng, John Bell, Baozhi Yu*, and Ying Ian Chen**

Y. Chen, X. Hu, Y. Zhu, Dr. Y. Fan, Dr. B. Yu, Prof. Y. I. Chen

Institute for Frontier Materials, Deakin University, 75 Pigdons Road, Waurn Ponds, Victoria Australia, 3216

E-mail: baozhi.yu@deakin.edu.au; ian.chen@deakin.edu.au

Dr. Y. Su

School of Engineering, STEM College, RMIT University, Melbourne, Victoria, Australia, 3000

Dr. M. Hong, Prof. J. Bell

Centre for Future Materials, University of Southern Queensland, Springfield, Australia, 4300

Prof. Z. Cheng

Institute for Superconducting and Electronic Materials, Australian Institute of Innovative Materials, University of Wollongong, Wollongong, New South Wales, Australia, 2500

**Material preparation**

Synthesis of Mn-Co-Fe oxides (MCF) nanoparticles: The MCF precursors were made up of 3 mmol FeCl_3_·6H_2_O, 1 mmol MnCl_2_·4H_2_O, and 1 mmol CoCl_2_·6H_2_O. CH_3_COONa (0.8 g), deionized water (3.5 mL), and ethanol (50 mL). The mixture was stirred for an hour to produce a homogeneous solution, and then it was poured into a 125 mL Teflon-lined stainless steel hydrothermal vessel. The vessel spent 12 hours in the oven at 180 °C. The resulting products were repeatedly cleaned with deionized water and ethanol after cooling to room temperature. After being dried for 12 hours at 60 °C in a vacuum oven, the MCF nanoparticles can be collected. The MCF nanoparticles with different ratios of Mn, Co, and Fe were obtained by adjusting the amount of FeCl_3_·6H_2_O, MnCl_2_·4H_2_O, and CoCl_2_·6H_2_O precursors. 4 mmol FeCl_3_·6H_2_O, 0.5 mmol MnCl_2_·4H_2_O, and 0.5 mmol CoCl_2_·6H_2_O were for MCF1; 3 mmol FeCl_3_·6H_2_O, 1 mmol MnCl_2_·4H_2_O, and 1 mmol CoCl_2_·6H_2_O were for MCF2; 2 mmol FeCl_3_·6H_2_O, 1.5 mmol MnCl_2_·4H_2_O, and 1.5 mmol CoCl_2_·6H_2_O were for MCF3.

**Material characterizations**

The crystal structures of catalysts were identified by XRD ('X'pert Powder, Panalytical Cu K radiation). The microstructures were observed using a field-emission scanning electron microscope (FE-SEM, SUPRA 55VP, ZEISS). Using a 300 kV-operating FEI Tecnai F30, images from transmission electron microscopy (TEM) and high-resolution TEM (HR-TEM) were generated. Energy-dispersive X-ray spectroscopy (EDS), high-angle annular dark-field scanning transmission electron microscopy (HAADF-STEM), and elemental mapping were performed on an FEI Talos F200X equipped with four electric refrigeration energy spectrum probes Super-X detectors operating at 200 kV. Metal components in MCFs were examined using inductively coupled plasma-optical emission spectroscopy (ICP-OES, Agilent 5800). Thermo Scientific's Nexsa Surface Analysis System was used to perform the XPS spectra.

**Electrochemical performance measurements**

The electrodes were prepared with MCF catalysts (50%), Ketjen Black (40%), and PVDF (10%). The NMP solvent (3 mL) was then filled with 50 mg of the mixture, and 12 hours were spent stirring. Next, carbon paper (CP, TGP-H-060, Toray) was placed on top of the resulting slurry. To get rid of the residual solvent, the produced electrodes were dried in a vacuum oven at 80 °C. To determine the mass of the active components on each CP wafer, the mass of each wafer was measured both with and without the catalyst. The catalyst was typically loaded between 0.4 and 0.6 mg cm^-2^.

The cathodes were constructed by combining them with lithium chips as the anode, a glass fiber separator (GF/C Whatman), and 1.0 M LiTFSI in TEGDME as the electrolyte, all within a glovebox filled with argon, ensuring oxygen levels remained below 0.1 ppm and moisture levels below 0.1 ppm. These cathodes were then incorporated into 2032-type coin cell cases that featured stainless-steel grid windows, allowing exposure to oxygen with a purity exceeding 99.5 % in one atmosphere. All measurements were conducted in a sealed test box (MSK-TE921) filled with oxygen at 25 ℃. The performance in terms of rate and cycling of the Li-O_2_ batteries was assessed using the LANHE (CT2001A) battery testing system. Additionally, cyclic voltammetry (CV) tests were performed using the electrochemical workstation (Solartron 1260, Solartron) at a scan rate of 0.5 mV s^-1^, covering a potential range from 2.2 to 4.5 V vs. Li/Li^+^.

**Simulation of magnetic field distribution and Li^+^ transport**

The magnetic field distribution of MCF electrodes after magnetization was simulated using COMSOL Multiphysics 6.0. As no currents are present, it is possible to model a permanent magnet using a scalar magnetic potential formulation. Three-dimensional models with a diameter of 2 μm and a space of 1 μm, 3*3*2 array for MCFs particles were built in this work as shown in Fig. S11 A. The *M*_s_ of MCFs were 75, 58, and 49 emu g^-1^, corresponding to MCF1, MCF2, and MCF3, respectively. Based on the simulated magnetic field results, the particle tracking module is introduced in COMSOL for calculating Li^+^ transport under an electric field and magnetic-electric coupled field. The release involves 10,000 particles each time, with a release interval of 0.1 ns. In total, 200 releases are conducted, and the particles are released in the downward direction along the Z-axis. The electric field voltage is set to 3.0 V. The results as shown in Fig. 4 e-h and Fig. S12-13 correspond to the 10th ns.

**Calculation methods**

Density functional theory (DFT) calculations were conducted using Vienna ab initio simulation package (VASP).^[1-3]^ The electron exchange-correlation was described by the functional of Perdew, Burke, and Ernzerhof (PBE) with generalized gradient approximation (GGA).^[4]^ The cutoff energy of 450 eV was used for the plane-wave basis. DFT-D3 method was employed to consider the van der Waals interactions.^[5]^ The vacuum layer was > 15 Å to prevent coupling between periodic slabs. The structures were relaxed until the total energy < 10^-5^ eV and the force on atoms < 0.01 eV Å^-1^. The structure includes 3 Mn atoms, 4 Co atoms, 25 Fe atoms, and 40 O atoms for MCF1.

The free energy of the intermediates in each step was calculated using the reference outlined method^[6]^

$$\Delta G=E-E_{0}+\Delta n_{Li}\left(\mu_{Li}-eU\right)+\Delta n_{O_{2}}\mu_{O_{2}}$$

in which *E* and *E*_0_ are the DFT calculated energy of the system at specific and initial steps. Δ*n*_Li_ and Δ*n*_O2_ are the adsorbed/desorbed numbers of Li and O_2_ for each step. *μ*_Li_ and *μ*_O2_ are chemical potentials of Li and O_2_. U is the electromotive force corresponding to the discharging (U_DC_), equilibrium (U_EQ_), and charging voltage (U_C_).

Adsorption energy (*E*_ads_) was calculated by

$$E_{\text{ads}}=E(\text{system})-E(\text{slab})-E(\text{molecular})$$

in which *E*(system) is the total energy with the adsorbed molecule, *E*(slab) is the energy of the bare slab, *E*(molecule) is the energy of isolated molecular


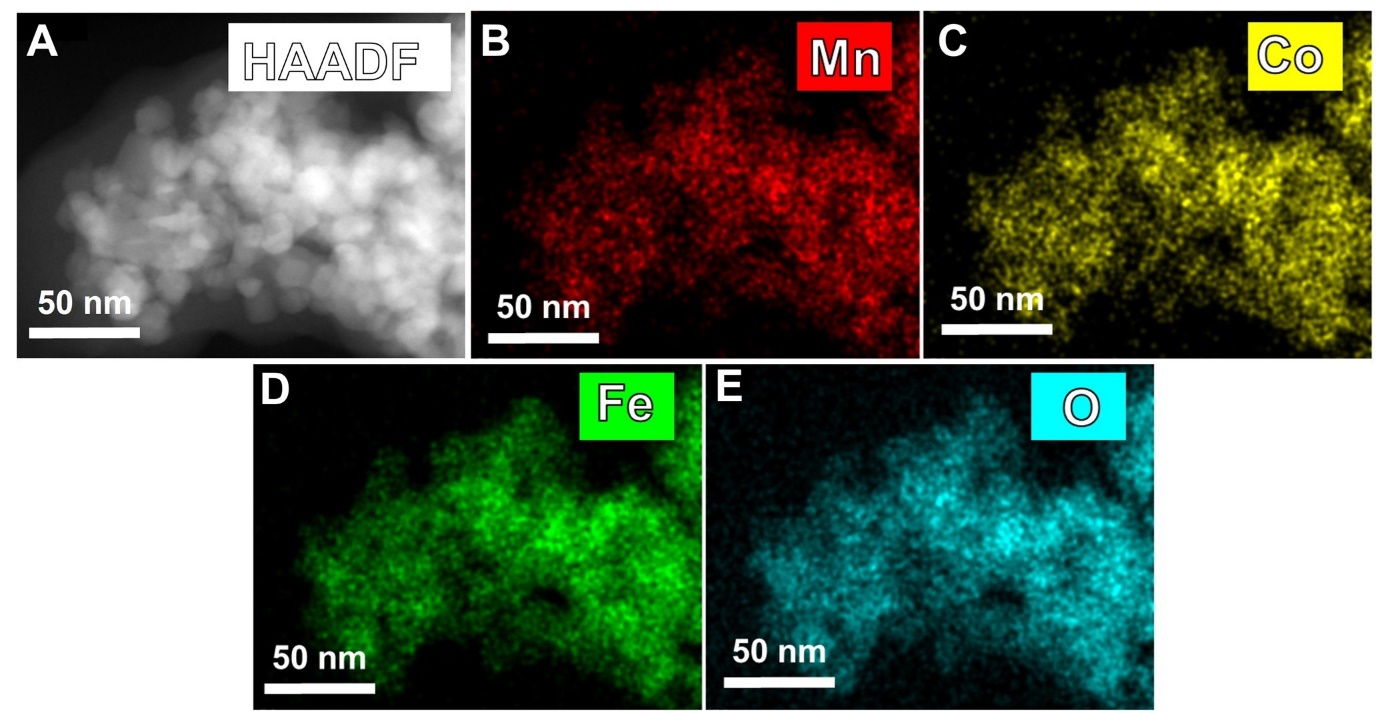


**Figure S1.** EDS mapping of MCF1 catalysts*
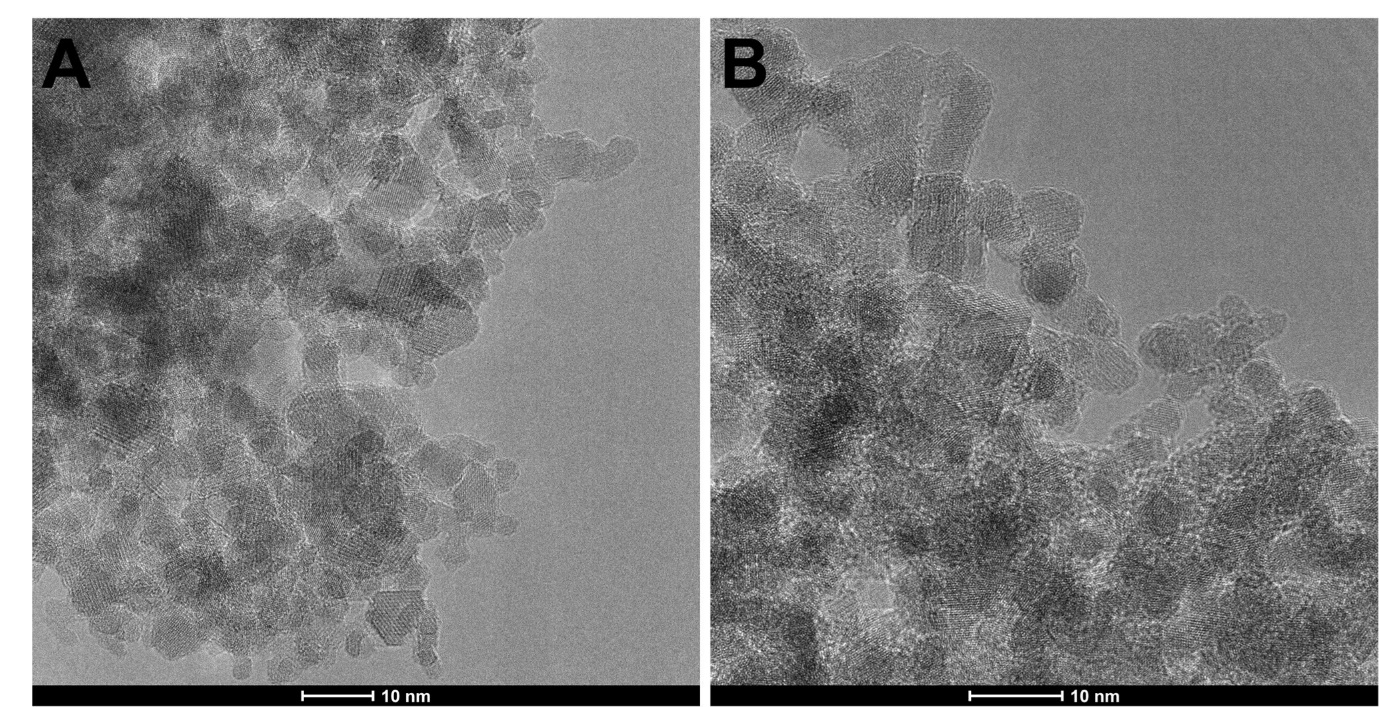
*

**Figure S2.** TEM images of (A) MCF2 and (B) MCF3


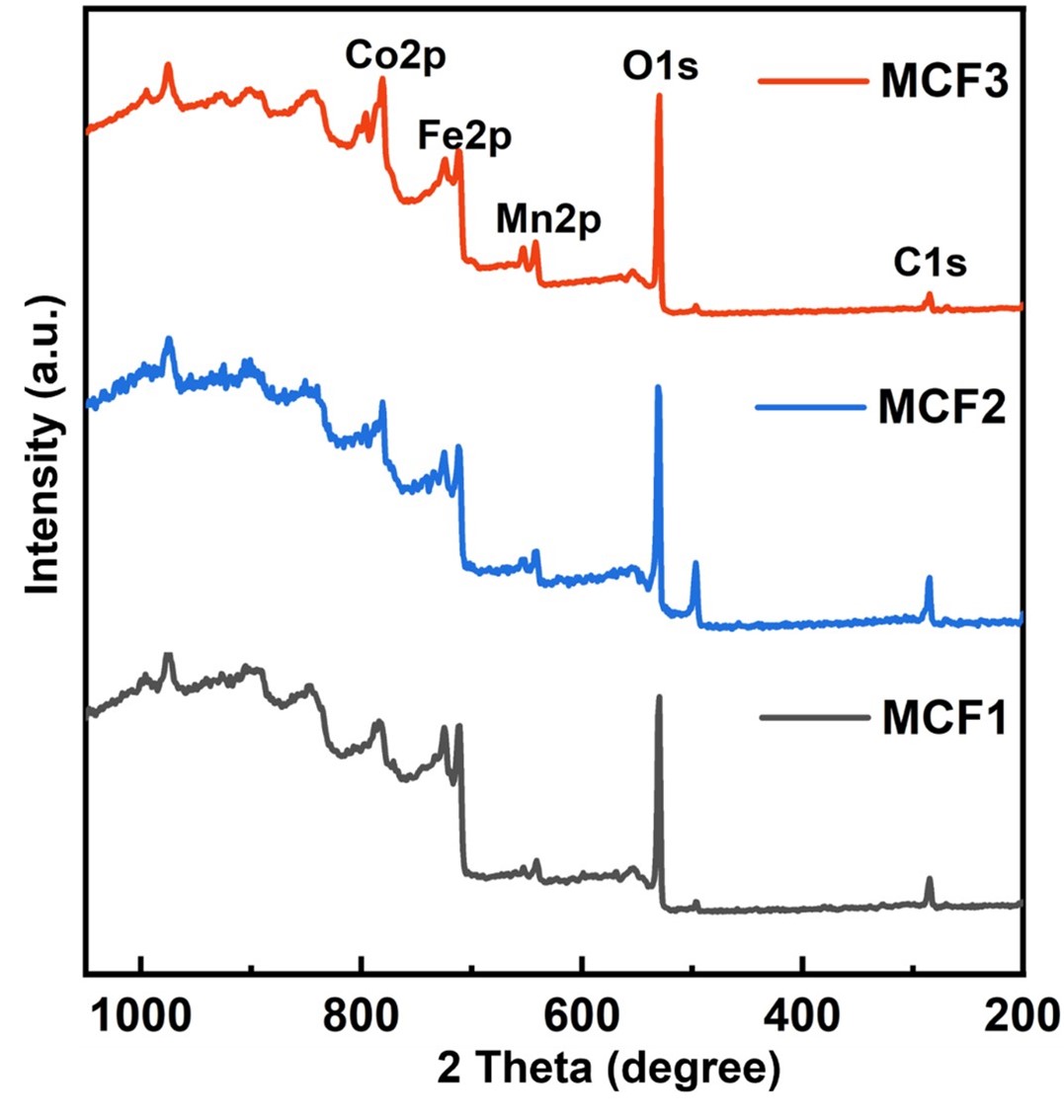


**Figure S3.** XPS survey spectra of investigated MCF


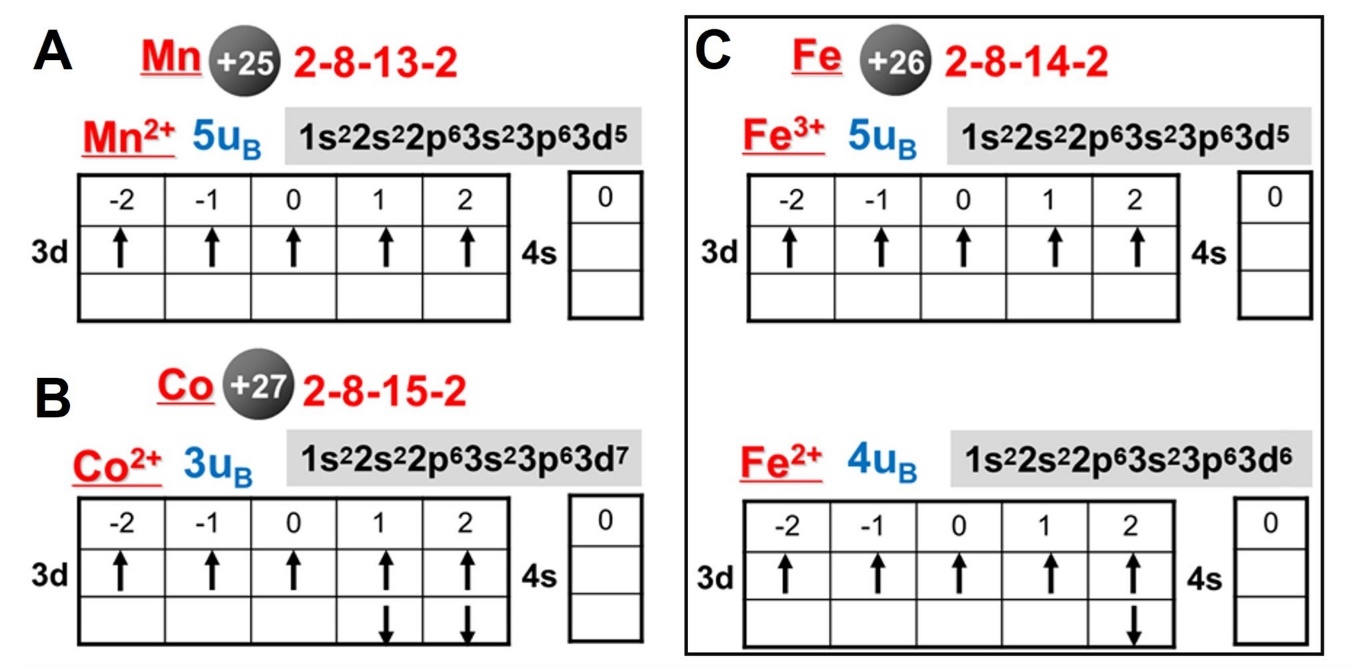


**Figure S4.** Electron configuration and number of Bohr magnetons of **(A)** Mn^2+^, **(B)** Co^2+^, and **(C)** Fe^3+^, Fe^2+^.


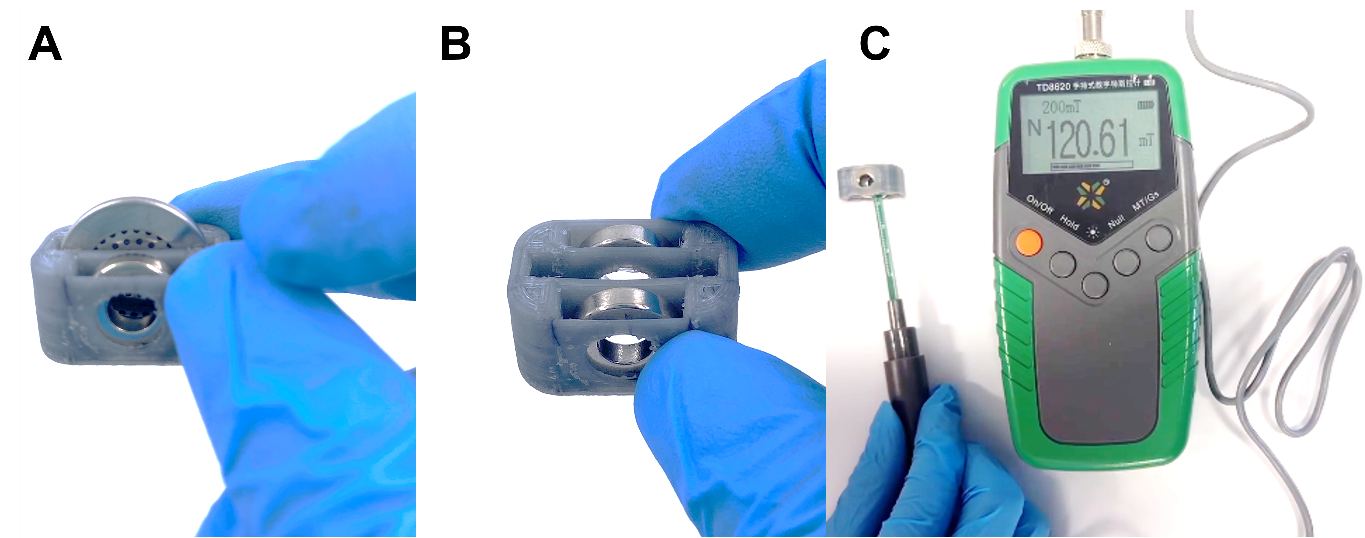


**Figure S5.** Optical photograph of **(A)** magnet and LOBs in a holder. The holder is made by 3-D printing to facilitate the strength of the magnetic field at the location of the battery **(B)** magnet and holder, **(C)** magnetic field strength on magnet center.


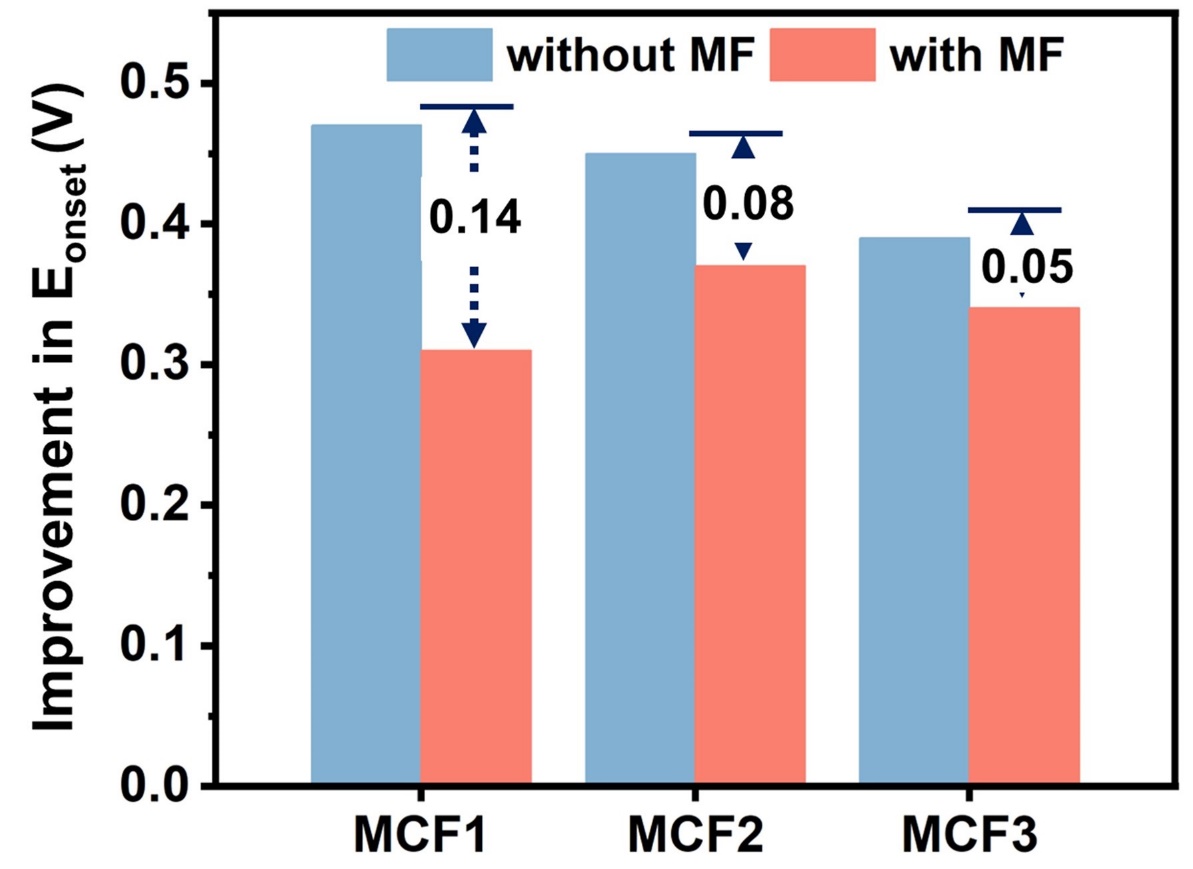


**Figure S6.** The onset potential changes of MCFs-catalysed LOBs by applying the magnetic field.


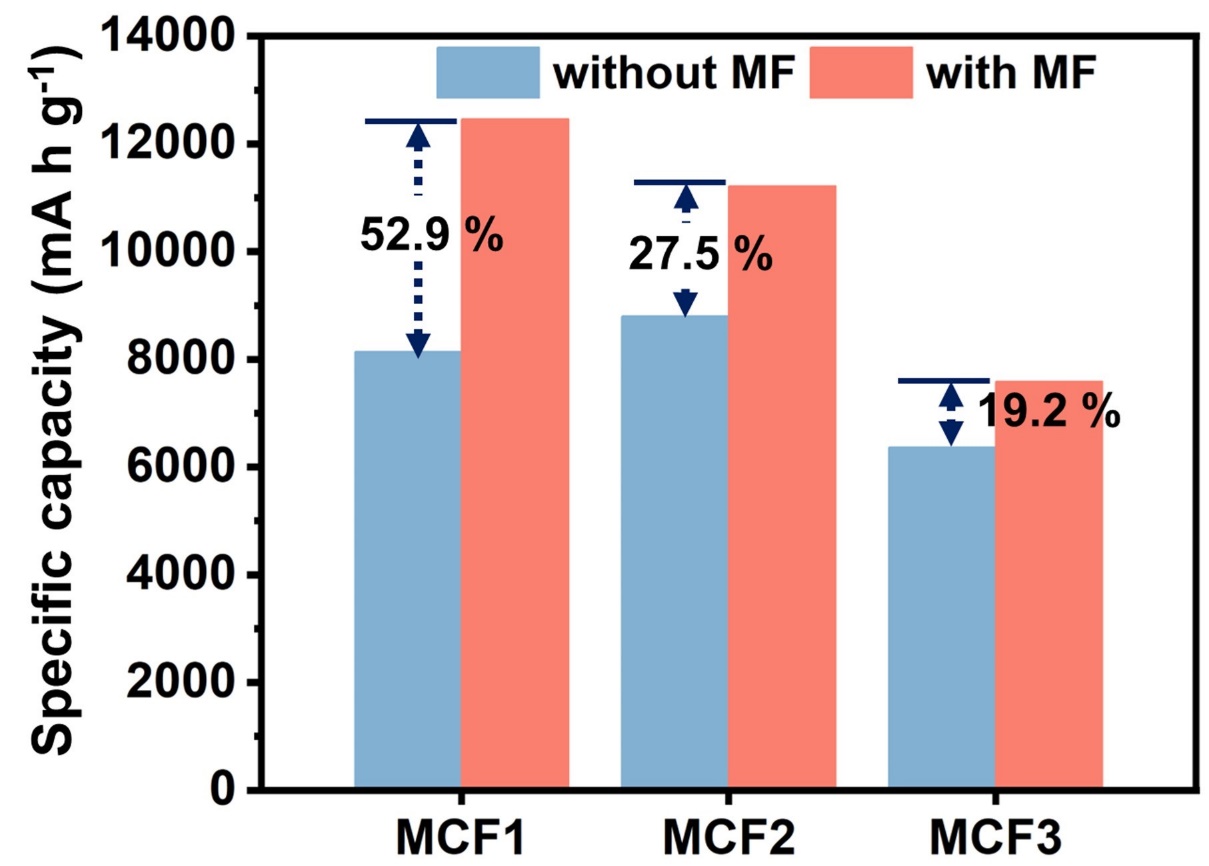


**Figure S7.** the specific capacity changes of MCFs-catalysed LOBs by applying the magnetic field.


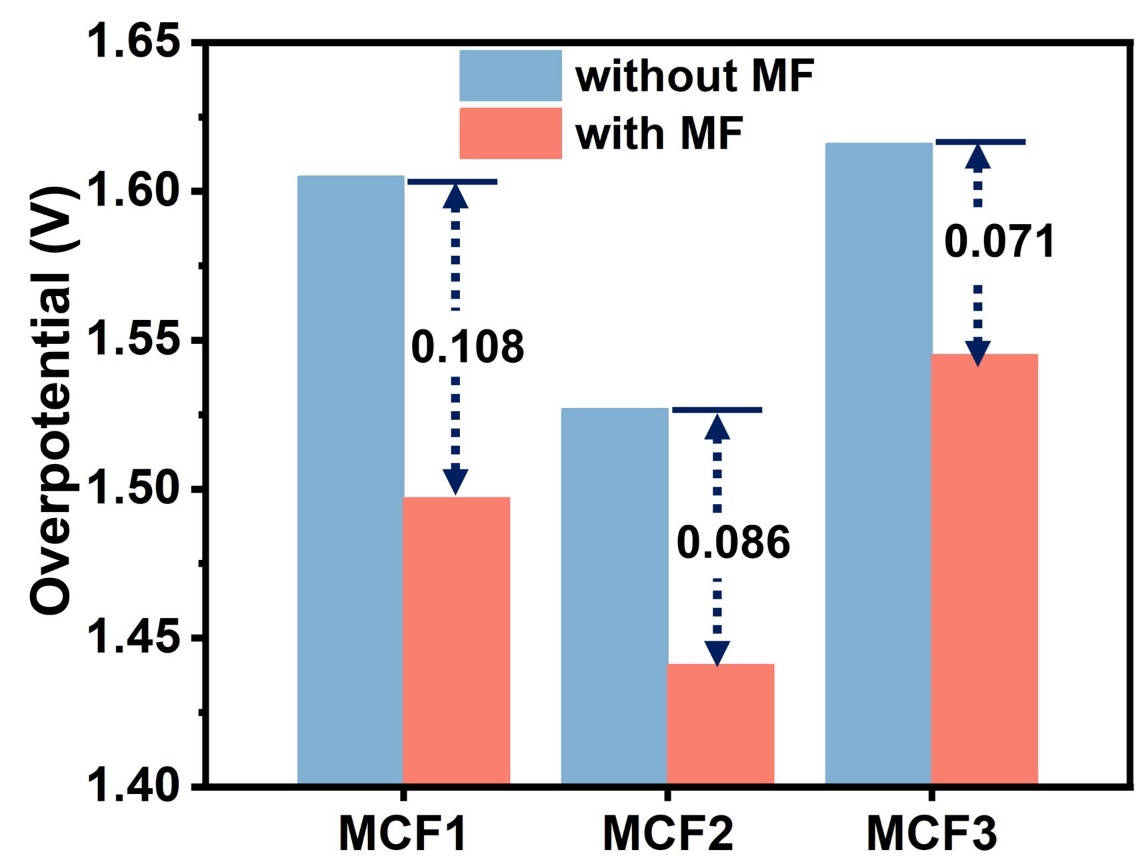


**Figure S8.** The overpotential changes of MCFs-catalysed LOBs by applying the magnetic field.

As shown in Figure S8, the overpotential of MCF1 catalyzed-LOBs decreased from 1.605 V to 1.497 V under the magnetic field. the MCF2 decreased from 1.527 to 1.441 V, and MCF3 decreased from 1.616 to 1.545 V. In the absence of an applied magnetic field, the MCFs catalyst exhibits its intrinsic catalytic performance, which is related to the doping ratio of Mn and Co elements. The main reason is that co-doping induces Mn to migrate to octahedral sites, optimizing the spatial distribution of Mn, which are the key active sites responsible for catalytic activity. It also increases the oxidation state of Mn, enabling stronger O_2_ adsorption and reducing the reaction energy barrier in key catalytic steps. An appropriate amount of Co does not dilute Mn active sites; instead, it enhances the intrinsic activity of individual Mn sites.^[7]^ These factors contribute to the low overpotential observed for MCF2. When a magnetic field is applied, the enhancement of the catalyst's performance depends on the material’s saturation magnetization (*M*_s_). The catalytic performance in the battery is a result of the synergy between the material and the magnetic field. Since MCF1 has the highest *M*_s_, it exhibits the greatest decrease in overpotential by 0.108 V, which can be attributed to the effect of the magnetic field. Nevertheless, despite having a lower *M*_s_ value than MCF1, MCF2 demonstrates a lower overpotential under the applied magnetic field, owing to its intrinsically superior catalytic performance.


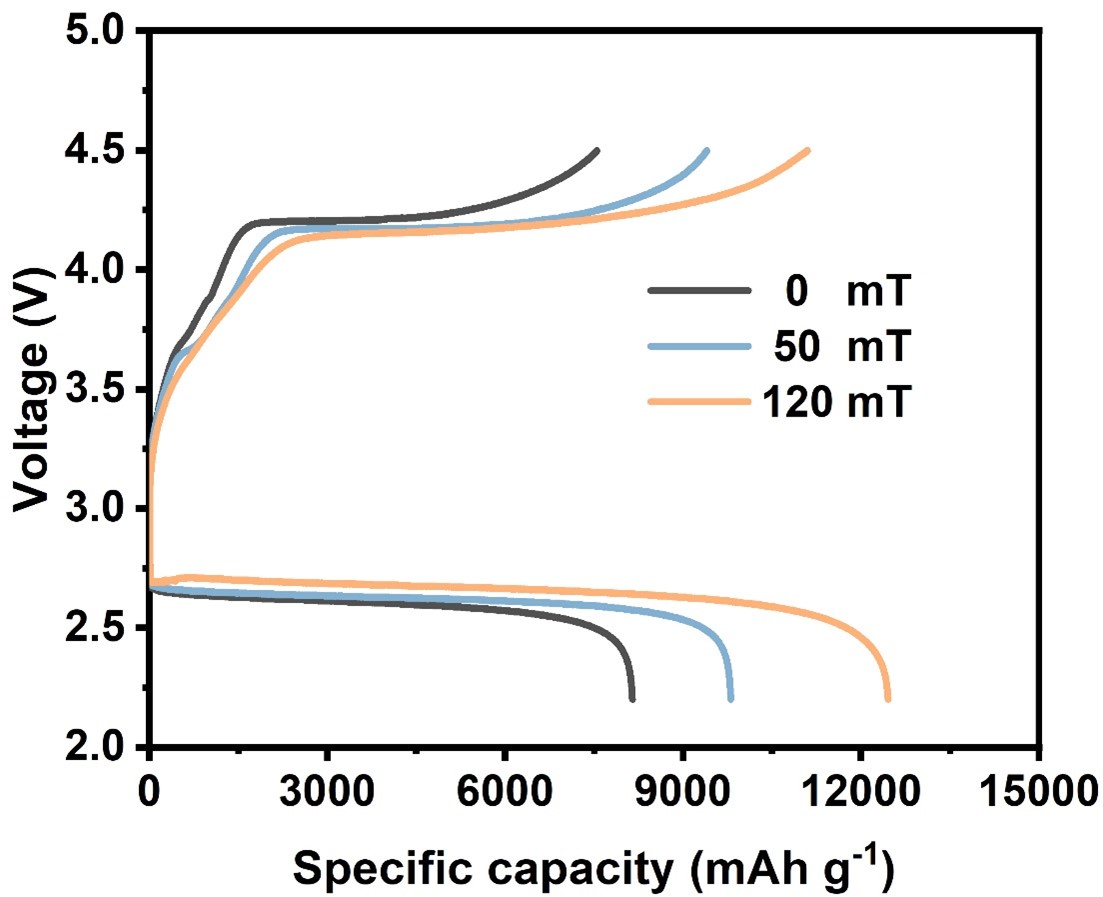


**Figure S9.** The full discharge/charge curves of MCF1 at 200mA g^-1^ under different magnet field intensities.

**
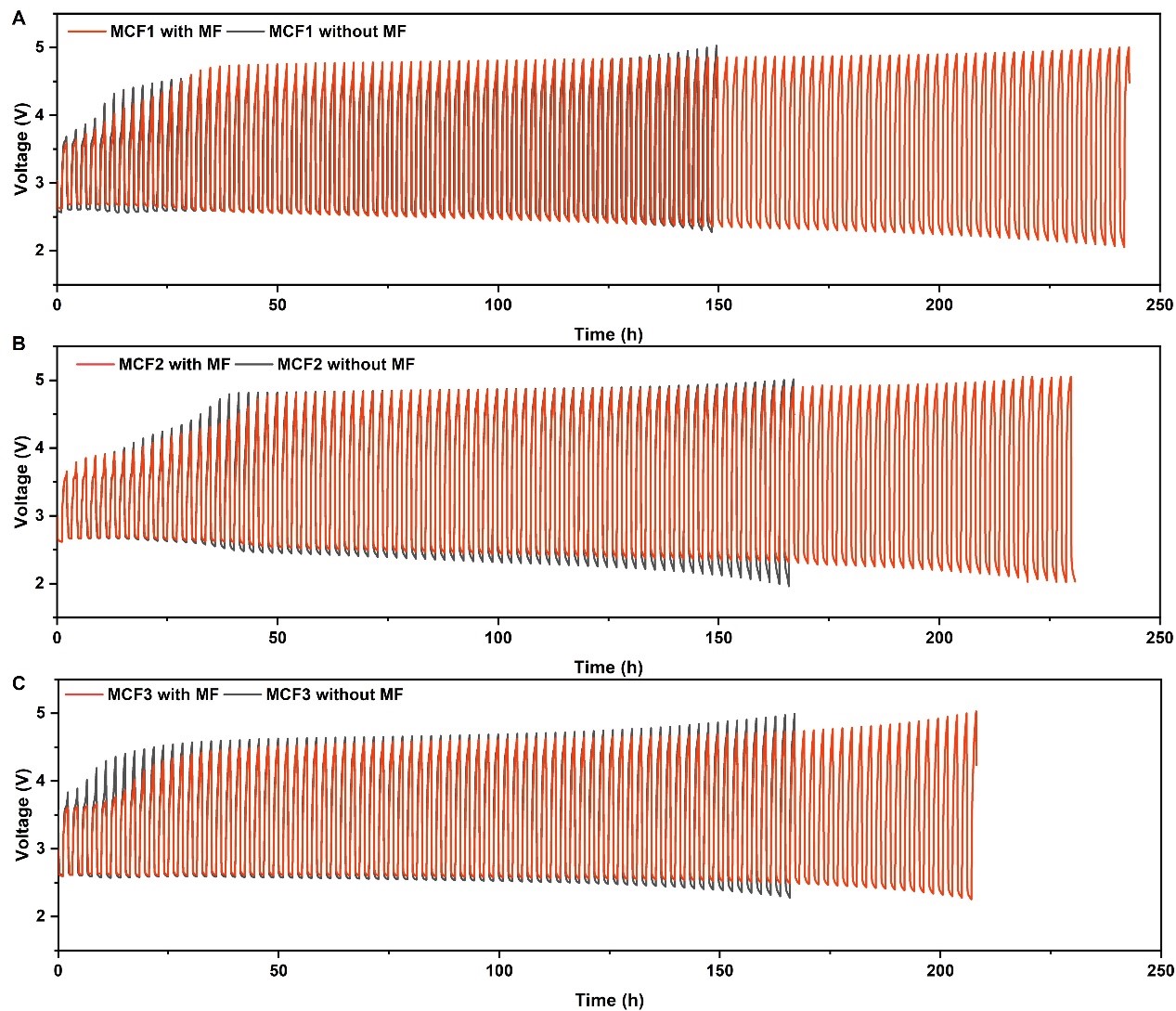
**

**Figure S10.** Cycling performance of **(A)** MCF1, **(B)** MCF2, and **(C)** MCF3 at 500 mA g^-1^ limited capacity at 500 mAh g^-1^.

The cycling performance of LOBs with and without a magnetic field is tested at 500 mA g^-1^ limited capacity of 500 mAh g^-1^. As shown in Figure S10A, the MCF1 catalyst exhibits a cycling stability close to 150 h without a magnetic field. When a magnetic field is applied, it increases to 243 h. Similarly, MCF2 increases from 167 to 230 h when a magnetic field is applied, and MCF3 increases from 167 to 208 h. By comparing the MCF catalysts, it can be observed that the cycling performance improves after the application of a magnetic field, with MCF1 showing the greatest improvement.

The effect of the magnetic field on the discharge product Li_2_O_2_ ( Figure S15, SEM images of the Li_2_O_2_ morphology), inhibiting lithium dendrite growth( Figure S16, SEM images of the Li anode after the charge), and improving electrode stability.


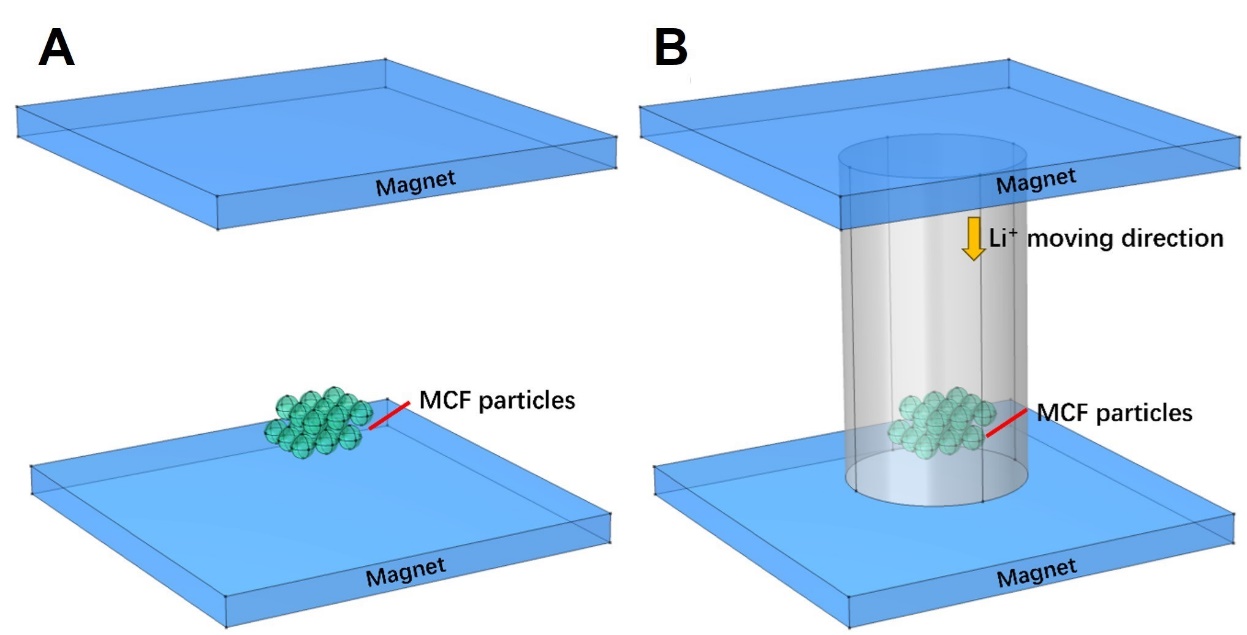


**Figure S11.** **(A)** The geometry model of the magnetic field distribution simulation of MCF electrodes after magnetization. **(B)** The geometry model of Li^+^ transport trajectory with the magnetic field.

In this study, we analyzed the magnetic field distribution of the magnetic particles after magnetization. This calculation can be conducted independently without considering the electrochemical processes. The movement of lithium ions in a battery is a complex electrochemical process. In this study, our focus is to investigate the influence of magnetic catalysts under an external magnetic field on the performance of lithium–oxygen batteries. Therefore, in our simulation, we primarily focused on the migration behavior of lithium ions in the electrolyte and their distribution on the electrode surface. Two scenarios were considered: one involving only the electric field, and the other involving coupled electric and magnetic fields. More complex electrochemical reaction kinetics and multiphase transport processes were not included. This simplification helps to isolate and highlight the effect of the magnetic field on the lithium-ion transport pathway and spatial distribution, thereby providing support and interpretation for the experimental observations.

In the simulation of the magnetic field distribution of MCF electrodes, we choose two layers of MCF particles to simplify the model, and each layer is a 3*3 array. The total number of MCF particles is 18. The MCF particles are built in a static magnetic field, which is provided by a pair of permanent magnets applied to both the cathode and the anode. The geometry model is shown in Figure S11A. For the simulation of Li^+^ transport trajectory with the magnetic field, based on the previous geometry model, we add the cylindrical region to further restrict the movement region of the Li^+^. The Li^+^ ions are released from the anode side and move to the cathode side, as shown in Figure 4d.

*
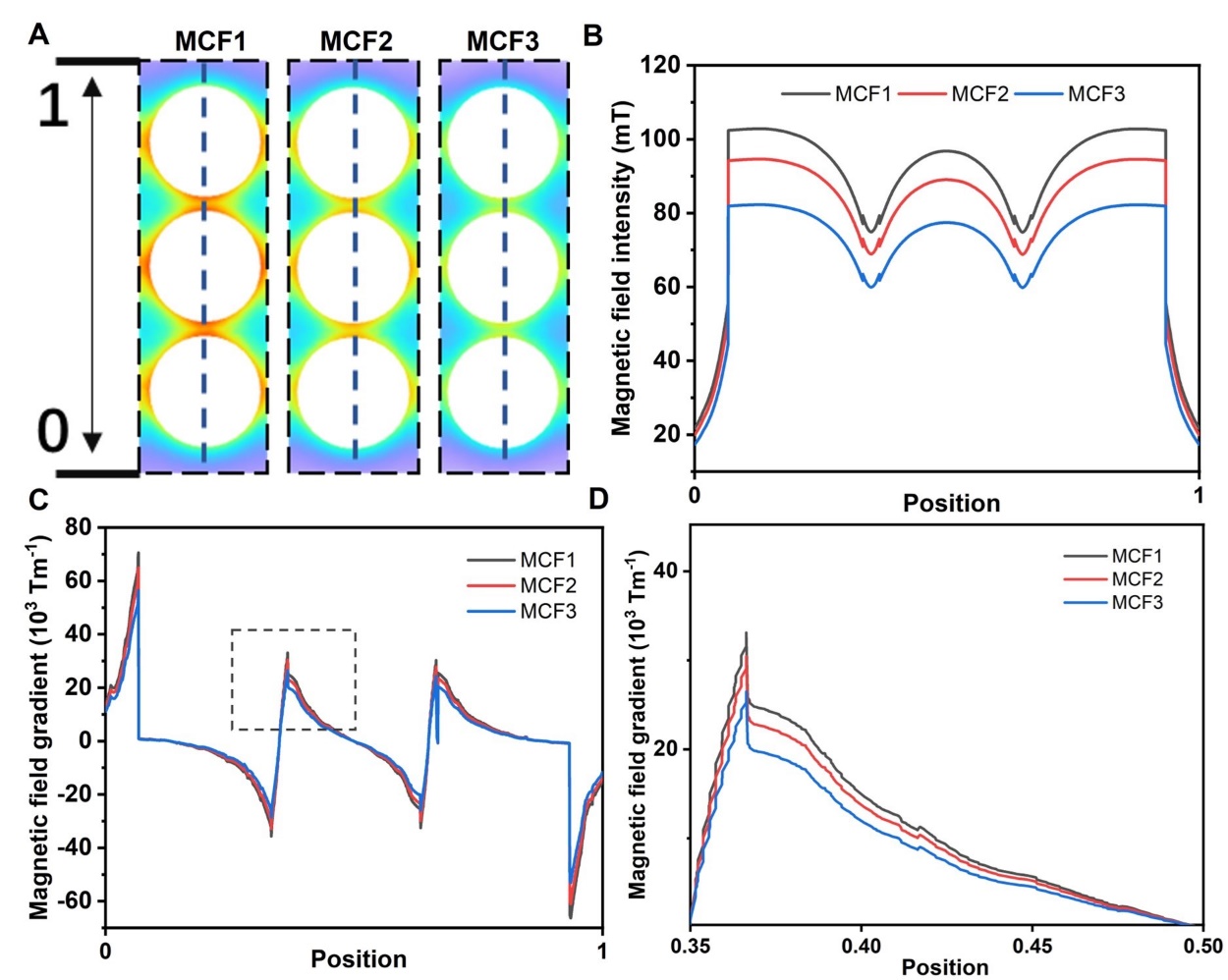
*

**Figure S12. (A)** Magnetic field intensity distribution of MCF particles. **(B)** Magnetic field intensity distribution on the central line. **(C)** Magnetic field gradient of MCFs. and **(D)** The enlarged area of the magnetic field gradient of MCFs.

We extracted the magnetic field distribution of MCFs from Figure 4B along the Y-axis. The length of the line is normalized with 0 as the starting point and 1 as the endpoint. The magnetic field intensity on the line is shown in Figure S12 B. The magnetic field intensity of MCF1 is the highest among these three MCF particles. Then, the magnetic field gradient (∇B) is calculated by using the data from Figure S12 B. As shown in Figure S12C, the value of ∇B around MCF1 particles is higher than the other two.


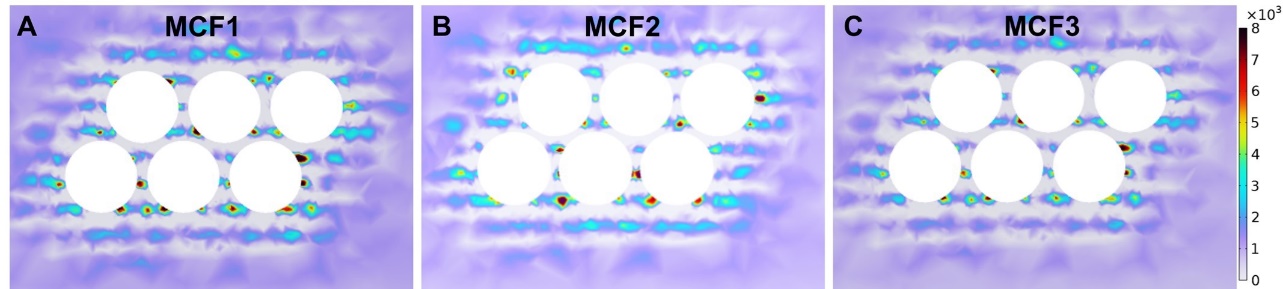


**Figure S13.** The Li^+^ transport trajectory with magnetic field simulation results on the XZ plane of MCFs **(A)** MCF1, **(B)** MCF2, and **(C)** MCF3.

*
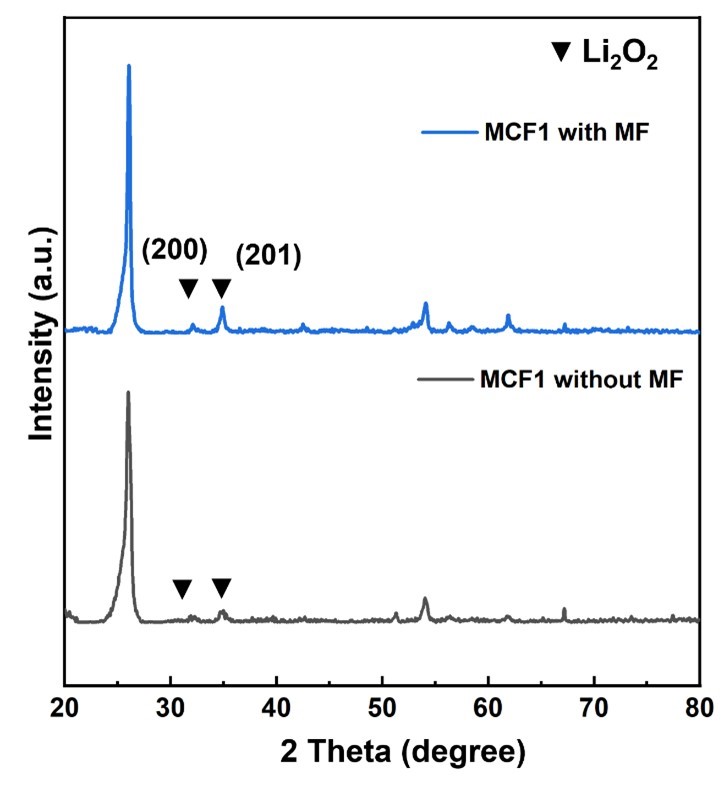
*

**Figure S14.** XRD patterns of MCF1-catalyzed LOBs after first discharge with and without magnetic field


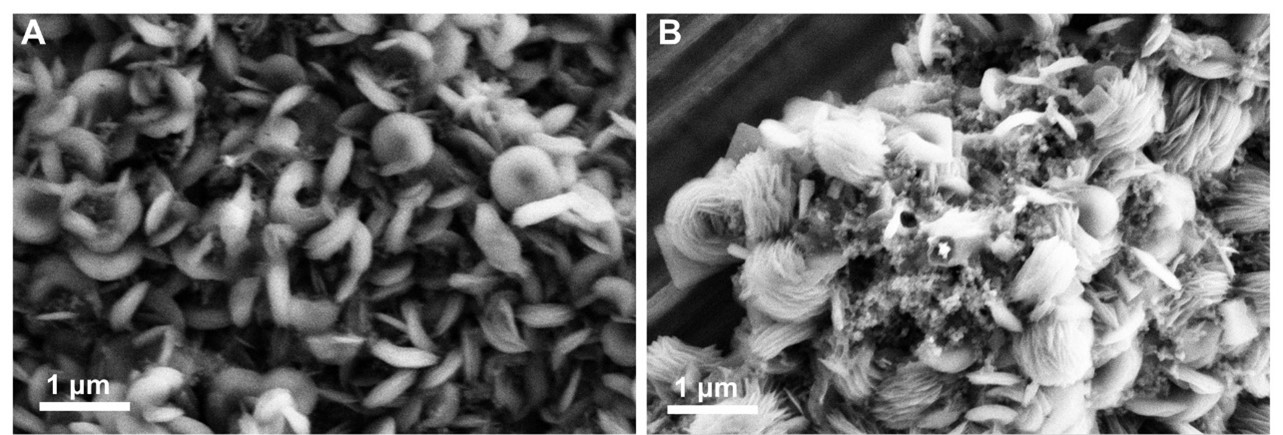


**Figure S15.** SEM images of the MCF cathode after fully discharged to 2.2 V at 200 mA g^-1^ **(A)** without a magnetic field and **(B)** with a magnetic field.

As shown in Figure S15, the discharge product of a lithium-oxygen battery without an applied magnetic field forms a toroidal (ring-like) structure, while the discharge product under the influence of a magnetic field consists of multiple nanosheets stacked in a cross pattern, forming a flower-like 3D structure. The flower-like Li_2_O_2_ has a larger specific surface area, which can improve the uniform distribution of the discharge products, thereby enhancing the capacity. The magnetic field can influence ion migration and the deposition process in the solution, promoting the growth of Li_2_O_2_ in a flower-like structure rather than a dense film or large particle structure. Due to its relatively loose structure, flower-like Li_2_O_2_ requires a lower overpotential for decomposition and is more easily oxidized compared to dense films or large particles. The lower overpotential not only improves energy efficiency but also reduces side reactions, enhancing cycling stability.^[8-9]^


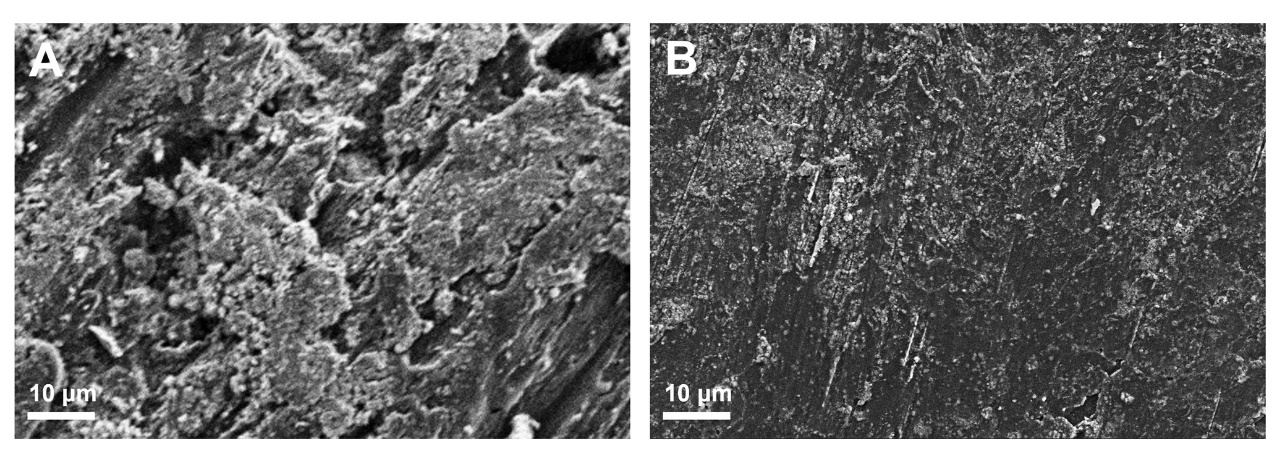


**Figure S16.** SEM images of the Li anode after fully recharged to 4.5 V at 200 mA g^-1^ **(A)** without a magnetic field and **(B)** with a magnetic field.


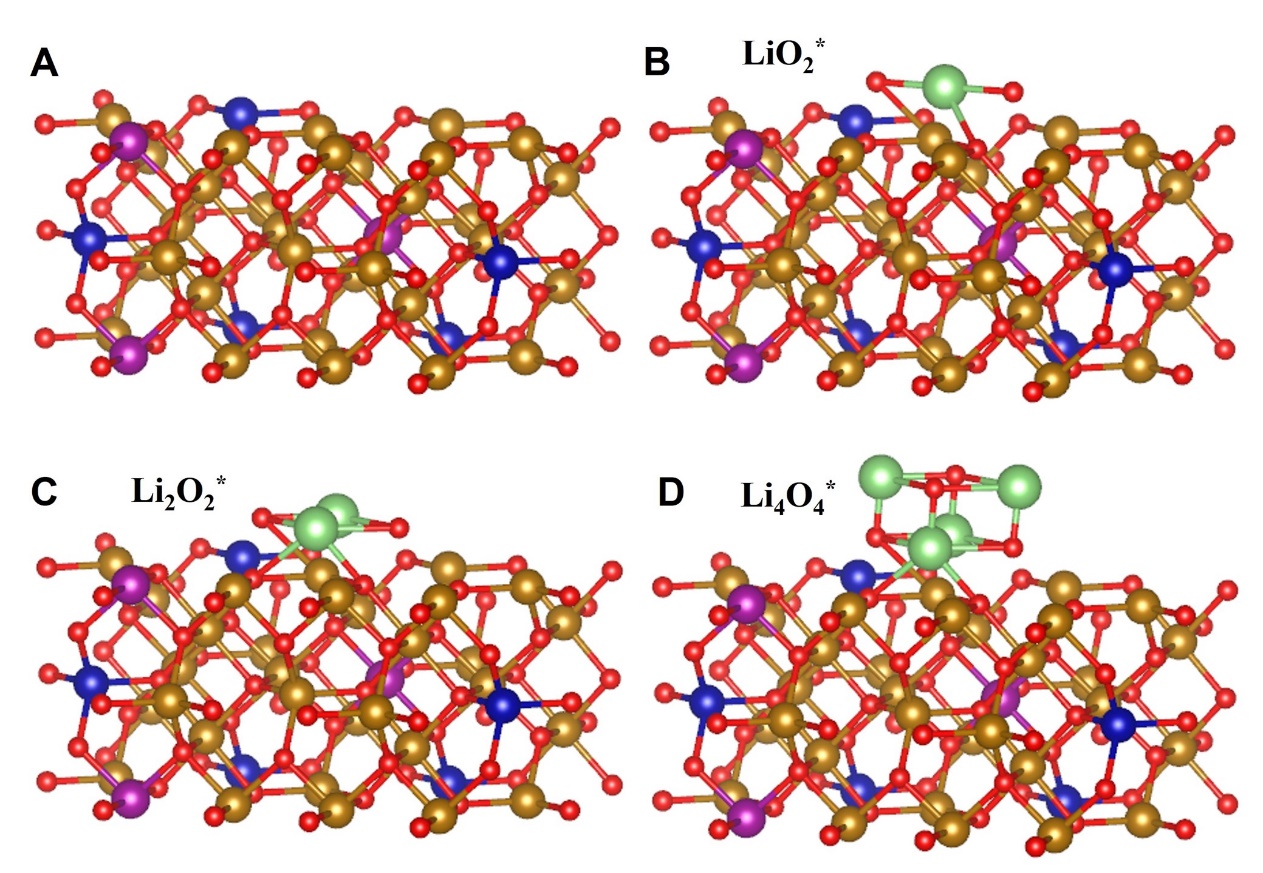


**Figure S17**. The adsorption structures of **(A)** MCF, MCF with **(B)** LiO_2_, **(C)** Li_2_O_2_, and **(D)** Li_4_O_4_.


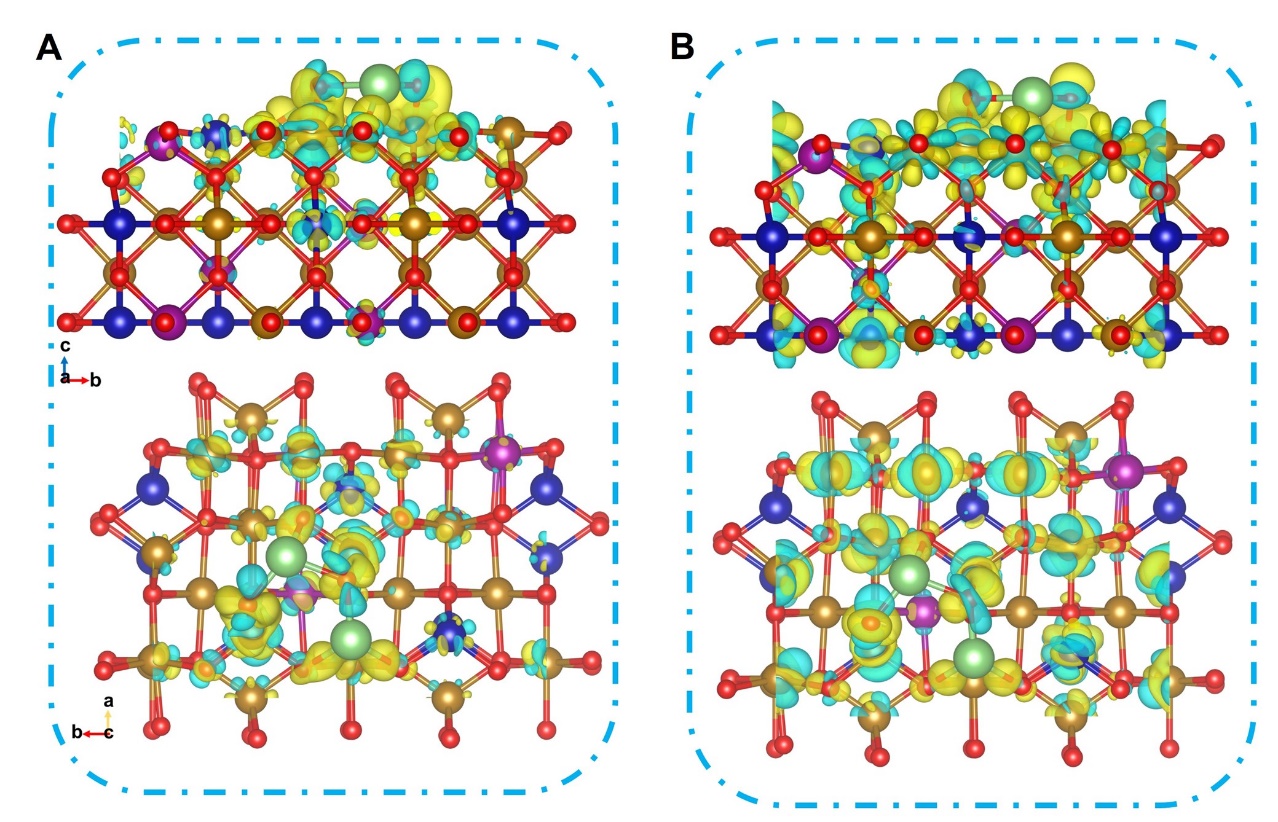


**Figure S18.** Charge density differences for Li_2_O_2_ absorption states **(A)** without magnetic field; **(B)** with magnetic field.

| **Parameter** | **Unit** | **Value** | **Description** |
| --- | --- | --- | --- |
| T_0_ | K | 298 | Temperature |
| M | A/m | 5E5 | Applied magnetic field intensity |
| Ms1 | emu/g | 75 | Saturation magnetization of MCF1 |
| Ms2 | emu/g | 58 | Saturation magnetization of MCF2 |
| Ms3 | emu/g | 49 | Saturation magnetization of MCF3 |
| R | μm | 2 | Diameter of MCFs catalysts |
| E0 | eV | 1 | Inital kinetic energy of Li^+^ |
| V | V | 3 | Electric field voltage |

**Table S1.** Simulation parameters used in this work

**Table S2.** Cycling performance and discharge capacity comparison of Li-O_2_ batteries with the other reported air cathode materials

| Catalysts | Cycling performance  Cycles @ 500 mA g^-1^ | Capacity  (mAh g^-1^) @ 200 mA g^-1^ | Ref. |
| --- | --- | --- | --- |
| CoMoO_4_ | 67 | 7706.2 | 10 |
| Fe-Co Oxide | 30 | 7953 | 11 |
| RuFe@NC | 93 (150 mA g^-1^) | 9186 | 12 |
| Fe@NC | 100 | ~8500 | 13 |
| CoNi-LDH | 123 (200 mA g^-1^) | 10644 | 14 |
| Co-SA-rGO | 40 | 12760.8 | 15 |
| MCF1 without MF | 75 | 8143 | This work |
| MCF1 with MF | 120 | 13416 |  |

**References**

[1] G. Kresse, J. Furthmüller, *Phys Rev B* **1996**, *54*, 11169-11186.

[2] J. Furthmüller, J. Hafner, G. Kresse, *Phys. Rev. B* **1996**, *53*, 7334-7351.

[3] G. Kresse, J. Furthmüller, *Comp Mater Sci* **1996**, *6*, 15-50.

[4] J. P. Perdew, K. Burke, M. Ernzerhof, *Physical Review Letters* **1996**, *77*, 3865-3868.

[5] S. Grimme, J. Antony, S. Ehrlich, H. Krieg, **2010**, *132*, 154104.

[6] H. R. Jiang, T. S. Zhao, L. Shi, P. Tan, L. An, *J. Phys. Chem. C* **2016**, *120*, 6612-6618.

[7] J. Kim, W. Ko, J. M. Yoo, V. K. Paidi, H. Y. Jang, M. Shepit, J. Lee, H. Chang, H. S. Lee, J. Jo, Adv. Mater. 2022, 34, 2107868.

[8] Z.-Z. Shen, C. Zhou, R. Wen, L.-J. Wan, *Journal of the American Chemical Society* **2020**, *142*, 16007-16015.

[9] C. Yao, X. Lei, C. Ma, Q. Zhang, X. Liu, Y. Ding, *Small* **2023**, *19*, 2301846.

[10] H. Zhou, L. Guo, R. Zhang, L. Xie, Y. Qiu, G. Zhang, Z. Guo, B. Kong, F. Dang, *Adv. Funct. Mater.* **2023**, *33*, 2304154.

[11] Q. Huang, B. He, W. Zhang, J. Wang, Y. Fan, X. Mai, Y. Wang, Y. Hou, Y. Du, P. Xie, F. Dang, *ACS Appl Mater Interfaces* **2020**, *12*, 30268-30279.

[12] J. Hong, S. Hyun, M. Tsipoaka, J. S. Samdani, S. Shanmugam, *ACS Catalysis* **2022**, *12*, 1718-1731.

[13] D. Li, J. Liang, S. J. Robertson, Y. Chen, N. Wang, M. Shao, Z. Shi, *ACS Appl Mater Interfaces* **2022**, *14*, 5459-5467.

[14] Y. Lin, Y. Zhang, J. Bao, J. Qiu, D. Guo, S. Zhang, M. Yuan, G. Sun, C. Nan, *Small* **2023**, *19*, 2302979.

[15] W. Zhang, J. Zheng, R. Wang, L. Huang, J. Wang, T. Zhang, X. Liu, *Small* **2023**, *19*, 2301391.
